# The somatic mutation landscape of the human body

**DOI:** 10.1186/s13059-019-1919-5

**Published:** 2019-12-24

**Authors:** Pablo E. García-Nieto, Ashby J. Morrison, Hunter B. Fraser

**Affiliations:** Department of Biology, Stanford University, 371 Jane Stanford Way, Stanford, CA 94305 USA

**Keywords:** Somatic mutation, Cancer, Aging, Genomic instability, Somatic evolution, Human

## Abstract

**Background:**

Somatic mutations in healthy tissues contribute to aging, neurodegeneration, and cancer initiation, yet they remain largely uncharacterized.

**Results:**

To gain a better understanding of the genome-wide distribution and functional impact of somatic mutations, we leverage the genomic information contained in the transcriptome to uniformly call somatic mutations from over 7500 tissue samples, representing 36 distinct tissues. This catalog, containing over 280,000 mutations, reveals a wide diversity of tissue-specific mutation profiles associated with gene expression levels and chromatin states. For example, lung samples with low expression of the mismatch-repair gene *MLH1* show a mutation signature of deficient mismatch repair. In addition, we find pervasive negative selection acting on missense and nonsense mutations, except for mutations previously observed in cancer samples, which are under positive selection and are highly enriched in many healthy tissues.

**Conclusions:**

These findings reveal fundamental patterns of tissue-specific somatic evolution and shed light on aging and the earliest stages of tumorigenesis.

## Background

In humans, somatic mutations play a key role in senescence and tumorigenesis [1]. Pioneering work on somatic evolution in cancer has led to the characterization of cancer driver genes [2] and mutation signatures [3]; the interplay between chromatin, nuclear architecture, carcinogens, and the mutational landscape [4–7]; the evolutionary forces acting on somatic mutations [8–11]; and clinical implications of somatic mutations [12].

Somatic mutations have been far less studied in healthy human tissues than in cancer. Early studies focused on blood [13, 14] as it is readily accessible and because of the known effects of immune-driven somatic mutation. Recently, somatic mutations have been characterized in tissues like the skin [15], brain [16, 17], esophagus [18, 19], and colon [20]. These studies confirmed that cells harboring certain mutations expand clonally, and the number of clonal populations—as well as the total number of somatic mutations—increases with age. Additionally, recurrent positively selected mutations in specific genes (e.g., *NOTCH1*) were observed. However, a more comprehensive understanding of somatic mutations across the human body has been limited by the small number of tissues studied to date.

Most studies on somatic evolution in healthy tissues have sequenced DNA from biopsies to high coverage. However, the transcriptome also carries all the genomic information of a cell’s transcribed genome, in addition to RNA-specific mutations or edits. RNA-seq has been used to identify germline DNA variants [21], and recently, single-cell (sc) RNA-seq was used to call DNA somatic mutations in the pancreas of several people [22].

To systematically identify somatic mutations in the human body and to investigate their distribution and functional impact, we developed a method that leverages the genomic information carried by RNA to identify DNA somatic mutations while avoiding most sources of false positives. We applied it to infer somatic mutations across 36 non-cancerous tissues, allowing us to explore the landscape of somatic mutations throughout the human body.

## Results

### Somatic mutation calling across 36 non-disease tissues from 547 people

**Fig. 1:**
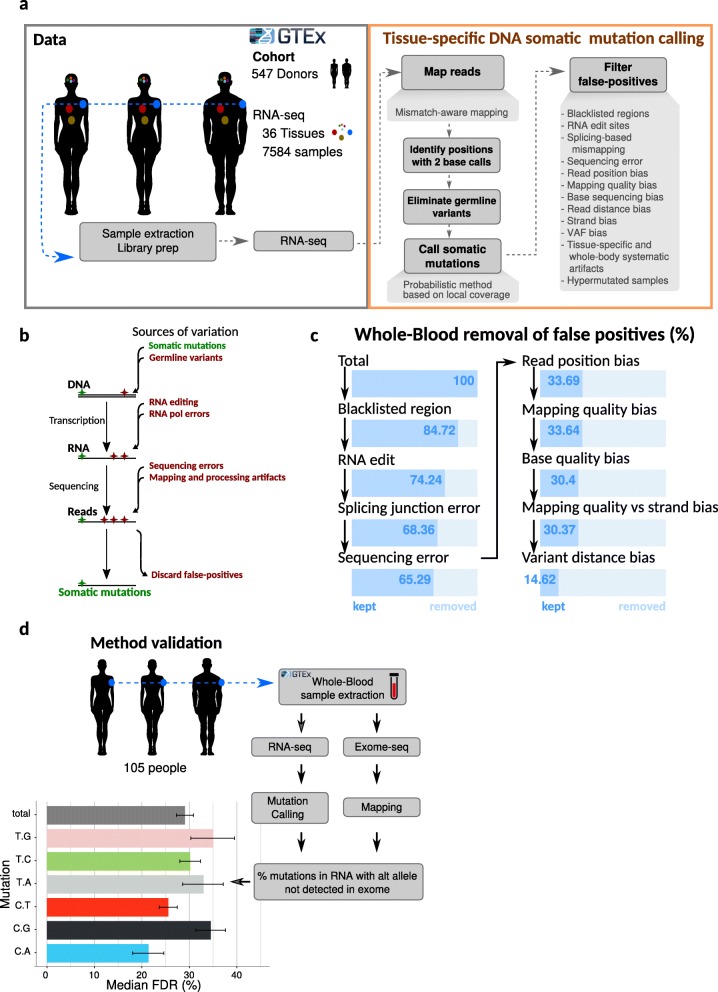
A method to identify DNA somatic mutations from RNA-seq. **a** A general overview of the method. RNA-seq reads were downloaded from GTEx v7 (left) and processed to identify positions with two different base calls at a high confidence. Then, sources of biological and technical artifacts were removed (right, see the “Methods” section). **b** Schematic illustrating potential sources of sequence variation. **c** Average percentage of variants detected in blood RNA-seq that are retained after each step of filtering (see the “Methods” section). **d** Validation of the method. For 105 individuals, we compared variant calls from exome DNA-seq data with those from RNA-seq of the same samples. Median FDR values per mutation type are shown, and they represent the fraction of mutations called in RNA-seq for which there are no exome reads supporting the same variant (see the “Methods” section and Additional file 1: Figure S1c). Error bars represent the 95% confidence interval after bootstrapping 10,000 times

Our method considers genomic positions where we observed two alleles in the RNA-seq reads and assesses whether they are likely to be *bona fide* DNA somatic mutations (Fig. 1a). We optimized the minimum levels of RNA-seq read depth, sequence quality, and number of reads supporting the variant allele to limit the impact of sequencing errors (Additional file 1: Figure S1a; see the “Methods” section). We then applied extensive filters to eliminate false positives from biological and technical sources, including RNA editing, sequencing errors, and mapping errors (see the “Methods” section; Fig. 1b, c; Additional file 1: Figure S2a; Additional file 2: Supplementary Tables legends and Additional file 3: Table S1).

To validate the method, we compared somatic mutation calls from 105 blood RNA-seq samples to exome DNA sequencing performed on the same samples (GTEx Consortium, 2017) (Fig. 1d). We observed a false-discovery rate (FDR) of 29% which represents the percentage of somatic mutations called from RNA-seq not having evidence in the corresponding DNA exome-seq sample (Fig. 1d, Additional file 1: Figure S1c; see the “Methods” section). Mutations with a higher variant allele frequency (VAF) had an overall lower FDR (Additional file 1: Figure S1d); accounting for this trend yielded an estimated 34% FDR for our complete set of mutation calls (see the “Methods” section). This is comparable to the 40% FDR in a previous study that inferred mutations from scRNA-seq in the pancreas [22].

After applying the pipeline and filters to RNA-seq data from the GTEx project, we retained a total of 7584 samples from 36 different tissues and 547 different individuals with no detectable cancer (Additional file 4: Table S2). This resulted in a total of 280,843 unique mutations (Additional file 5: Table S3), most of which were rare across samples (median frequency = 0.026% of samples; Additional file 1: Figure S2b) and across individuals (median frequency = 0.37% of individuals; Additional file 1: Figure S2c). We found no enrichment of mutations with VAF close to 0.5 (Additional file 1: Figure S1e), suggesting that there is little contamination of heterozygous germline variants.

**Fig. 2:**
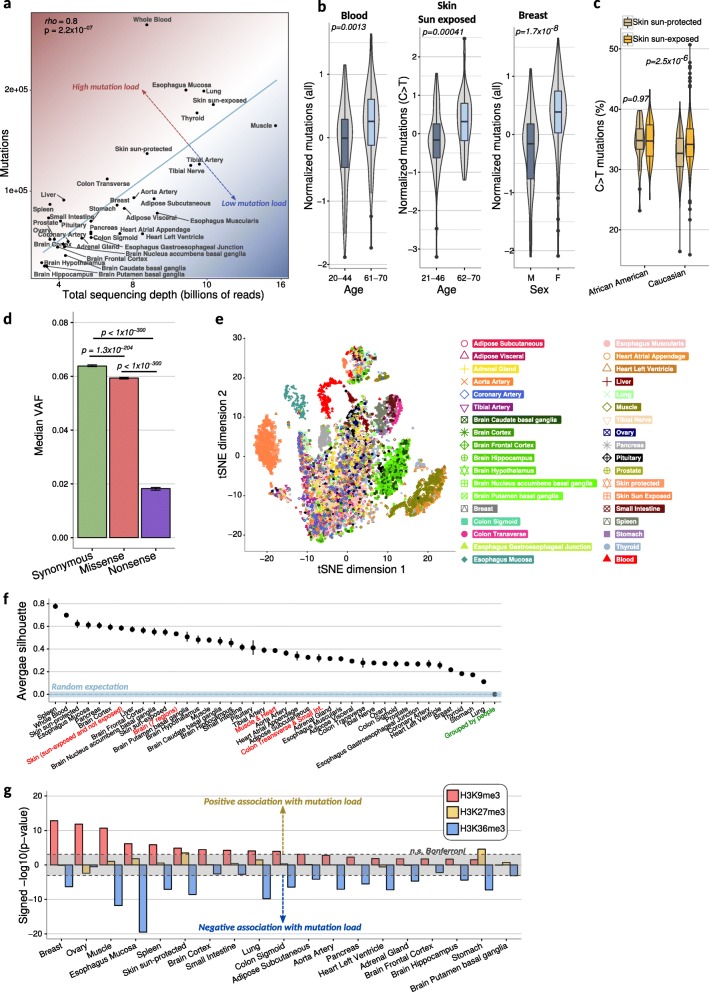
Cross-tissue analysis of somatic mutations. **a** The total number of mutations observed in a tissue depends on the sequencing depth of that tissue. Sequencing depth is defined as the cumulative amount of uniquely mapped reads across all samples of a tissue. A linear regression line is shown in blue; tissues above it exhibit more mutations than expected by sequencing depth, while tissues below it show fewer mutations than expected. *Rho* is the Spearman coefficient. **b** Examples of significant mutation associations with age and biological sex (see Additional file 1: Fig. S4 and Additional file 6: Table S4 for all tissue data). Age ranges represent the youngest and oldest quartiles for each tissue. To control for sequencing depth and other technical artifacts, mutation values were obtained as the residuals from a linear regression (see the “Methods” section). *p* values are from a two-sided Mann-Whitney test. **c** Caucasian sun-exposed skin shows a higher percentage of C>T mutations compared to the sun-protected skin, while no such difference was seen for African-American skin. *p* values are from two-sided Mann-Whitney tests. **d** Median variant allele frequency (VAF) for each mutation type based on their impact to the amino acid sequence; error bars represent the 95% confidence interval after bootstrapping 1000 times; *p* values are from two-sided Mann-Whitney tests. **e** tSNE plot constructed from a normalized pentanucleotide mutation profile (the mutated base plus two nucleotides in each direction; see the “Methods” section for normalization details) and all samples in this study. **f** Average silhouette scores representing the coherence of selected groups of samples from the tSNE space in panel **e**; a score of 1 represents maximal clustering, whereas 0 represents no clustering (see the “Methods” section). Grouping was performed by tissue-of-origin, or multiple tissues combined (red labels). “Grouped by people” (green label) is an average silhouette score after grouping samples by their person-of-origin from 20 randomly selected people. The blue dashed line represents the average random score expectation after permuting tissue labels (see the “Methods” section), and the blue stripes are ± two standard deviations. Error bars in points represent the 95% confidence interval based on bootstrapping 10,000 times. **g** Mutation load is positively associated with H3K9me3 and/or negatively associated with H3K36me3 across most tissues analyzed. *p* values were obtained from a linear regression using all histone modifications as explanatory variables (see the “Methods” section). Gray range denotes non-significant *p* values after Bonferroni correction

We first investigated the factors influencing mutation counts per sample and tissue (see the “Methods” section). The main contributor was sequencing depth and to a lesser extent other biological and technical factors (Additional file 1: Figure S2d, Additional file 6: Table S4). Tissues that have more mutations than expected from sequencing depth include those most often exposed to environmental mutagens or with a high cellular turnover like the skin, lung, blood, esophagus mucosa, spleen, liver, and small intestine (Fig. 2a). On the other end of the spectrum are those with low environmental exposure or low cellular turnover such as the brain, adrenal gland, prostate, and several types of muscle—heart, esophagus muscularis, and skeletal muscle (Fig. 2a).

We observed similar trends across all six possible point mutation types (complementary mutations—such as C>T and G>A—were collapsed because when one is detected the other is always present on the other DNA strand) (Additional file 1: Figure S3a). C>T and T>C mutations were the most abundant types, followed by C>A (Additional file 1: Figure S3b; Additional file 7: Table S5).

### Mutation load is affected by age, sex, ethnicity, and natural selection

After controlling for all known technical factors (see the “Methods” section), we observed that age was positively correlated with mutation load across most tissues (Additional file 1: Figure S4a; 29/36 tissues with Spearman *ρ* > 0, binomial *p* = 1.6 × 10^−4^). Blood showed the most significant age association for all mutation types combined (Fig. 2b; 1st–4th-quartile Wilcoxon *p* = 0.013; Spearman *ρ* = 0.21, Benjamini-Hochberg [BH] FDR < 0.0001), whereas the sun-exposed skin was the most significant for C>T mutations, the most common mutation associated with UV radiation (1st–4th-quartile Wilcoxon *p* = 0.00041; Spearman *ρ* = 0.17 BH-FDR = 0.02). We observed other strong age associations in several brain regions, and in particular the basal ganglia (Additional file 1: Figure S4a), supporting the hypothesis that somatic mutation load could contribute to the increasing risk of neurodegenerative diseases with age [23].

In addition to age, other biological factors also contributed to somatic mutations. For example, women show a much greater mutation load in breast than men (Fig. 2b; Wilcoxon *p* = 1.7 × 10^−8^). We also observed female-biased mutations (BH-FDR < 0.1) in the subcutaneous adipose, visceral adipose, liver, and adrenal gland; in contrast, we found no significant male-biased mutations after multiple hypothesis correction (Additional file 1: Figure S4c; Additional file 8: Table S6). Ethnicity can also affect mutation rates: we found a significant increase of C>T mutations in Caucasian sun-exposed skin compared to non-exposed skin, but no corresponding difference in African-Americans (Fig. 2c, Additional file 1: Figure S4b), likely due to protection against UV radiation provided by higher melanin content. Finally, we observed that the number of stem cell divisions [24] in a tissue was weakly correlated with mutation load (Additional file 9: Note S1, Additional file 1: Figure S5).

To investigate the role of natural selection on somatic mutations, we examined the variant allele frequency (VAF) within every sample where a given mutation was observed. We expected that most mutations we observed were present in clonal expansions of cells, since mutations only present in a single cell would be too rare for our method to detect; therefore, VAFs reflect the extent of this expansion, which can be affected by natural selection on cellular proliferation rate as well as other factors such as how long the clone has been proliferating. In general, we expect synonymous mutations that do not change the amino acid sequence to have the weakest effects on cellular fitness, missense variants that change an amino acid to have intermediate effects, and nonsense mutations that introduce a premature termination codon to have the strongest effects. These mutations could then show differing VAFs according to how likely they are to affect (and most likely reduce) cellular fitness, with stronger deleterious effects leading to lower VAF. Indeed, this was the pattern we observed, with synonymous variants having significantly higher VAF than both missense and nonsense variants (Fig. 2d).

Since our mutations are called from the transcriptome, these results could be influenced by nonsense-mediated decay (NMD), which degrades transcripts containing premature termination codons. While we did observe evidence of NMD (Additional file 1: Figure S4d), we found that nonsense mutations in the last exon of genes—which are not subjected to NMD [25]—still have significantly lower VAF compared to synonymous and missense mutations (Wilcoxon *p* = 1.9 × 10^−213^ and *p* = 5.6 × 10^−150^, respectively; Additional file 1: Figure S4d), confirming the existence of negative selection against nonsense mutations. Therefore, in contrast to what has been observed in cancer [9], we found evidence of negative selection acting against both missense and nonsense mutations.

### Somatic mutation profiles are tissue-specific and associated with chromatin state

To visualize the tissue specificity of somatic mutational patterns, we applied t-SNE [26] to the full set of mutations called in each of the 7584 tissue samples (including the 2-bp flanking each mutation; see the “Methods” section; Fig. 2e). We then used a silhouette score (SS) to quantify clustering of samples in this two-dimensional space (see the “Methods” section), where a value *SS* = 1 indicates samples that are maximally clustered within a group and a value *SS* = 0 means that samples are equidistant to samples within vs outside a group. Grouping samples by their donor-of-origin results in a mean *SS* = 0, no different than expected by chance (Fig. 2f). However, grouping samples by tissue resulted in values 0.7 *> SS* > 0.1, suggesting tissue-specific mutation patterns. The spleen, blood, skin, liver, and esophagus mucosa exhibited the most coherent profiles (Fig. 2f, Additional file 1: Figure S6a, d); conversely, the artery, lung, stomach, and thyroid had the least consistent profiles (Fig. 2f, Additional file 1: Figure S6e, f). Interestingly, some tissues cluster together as shown by SSs obtained by grouping samples from more than one tissue, suggesting shared mutagenic or repair processes (Fig. 2f; Additional file 1: Figure S6b, c; e.g., skeletal muscle and heart, *SS* = 0.38; seven brain regions, *SS* = 0.53; colon and small intestine, *SS* = 0.31). These results suggest that the somatic mutation landscape of the transcribed genome is largely defined by tissue-of-origin.

To explore the source of this tissue specificity, we hypothesized that chromatin may play an important role, as it does in cancer [4, 5]. We assessed the association between tissue-specific mutation rates and five histone modifications measured across many human tissues by the Roadmap Epigenomics Project [27] (Additional file 10: Table S7; see the “Methods” section). Across most tissues (except the brain), there is a strong positive association between mutation rate and a marker for heterochromatin (H3K9me3); conversely, we found strong negative associations with actively transcribed regions (H3K36me3; Fig. 2g; Additional file 1: Figure S4e). These results suggest that chromatin associations with mutation rates in the human body are nearly ubiquitous and arise prior to cancer development.

### Mutational strand asymmetries are widespread and vary across individuals

Cancer mutations often occur preferentially on one DNA strand, either in reference to transcription or to DNA replication (leading vs lagging strand) [28]. Since our mutations are derived from transcribed exons, we focused on transcriptional mutational strand asymmetries.

**Fig. 3:**
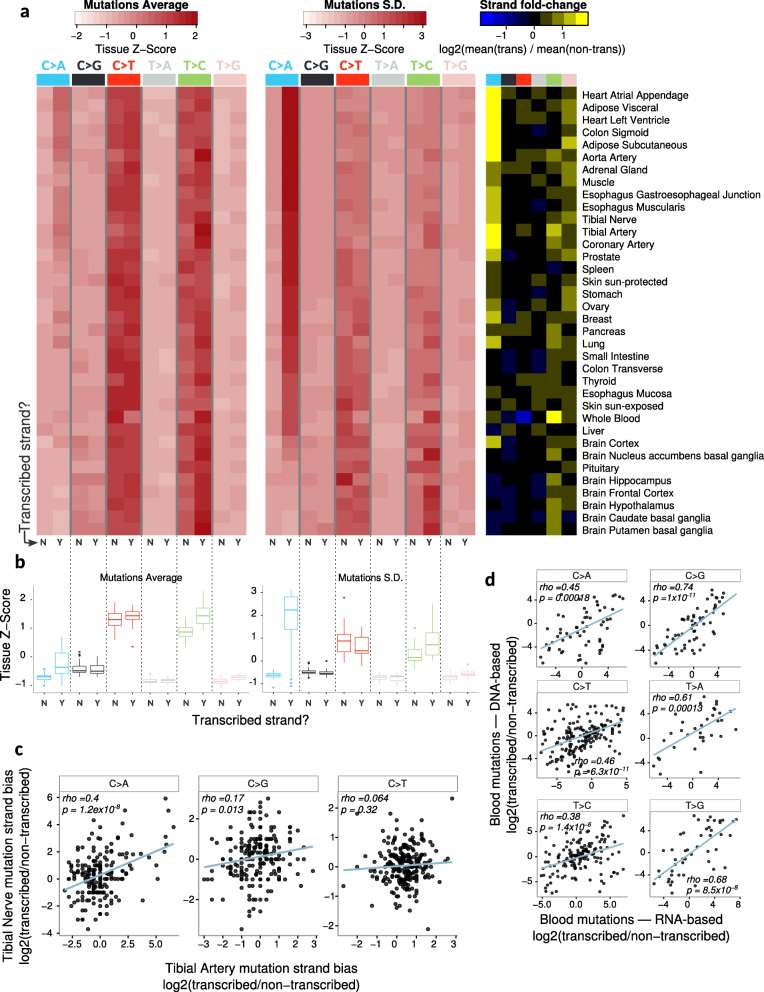
Somatic mutational strand asymmetries. **a** Mutation average and S.D. for each strand with respect to transcription (left and middle panels) and the ratio of mean mutations on the transcribed over the non-transcribed strand (right panel). **b** Distribution of *z*-scores for mutation averages and standard deviations on each strand (from panel **a**) across all samples. **c** Example of intra-individual correlation of C>A strand asymmetry in two tissues; each point represents an individual for which we generated mutation maps in the two tissues (see Additional file 1: Figure S7a for all tissue pairwise comparisons). **d** Correlation of mutational strand biases between calls from RNA-seq and matched DNA-seq; each point represents the mutation strand bias observed in one gene across all 105 blood samples. Blue lines in all scatter plots are based on a linear regression; *rho* values are the Spearman correlation coefficients

We observed the strongest asymmetry for C>A mutations, which preferentially occur on the transcribed strand in most tissues except for the brain (Fig. 3a, b; mean [transcribed/non-transcribed] ratio = 1.6 in the non-brain, 0.98 in the brain). C>A mutation load on the transcribed strand also showed the greatest variation between individuals (Fig. 3a, b). C>A asymmetries were often correlated between different tissues of the same person (Fig. 3c, Additional file 1: Figure S7a), suggesting a common factor can induce C>A mutations specifically on the transcribed strand (or equivalently, G>T mutations on the non-transcribed strand) across many of an individual’s tissues. This factor is of unknown origin; it could be intrinsic (e.g., genetic), extrinsic (e.g., exposure to a mutagen), or a combination of the two. Interestingly, this same C>A asymmetry has been observed in lung and ovarian cancer [28] and in cell lines exposed to different environmental agents [29]; our results suggest it is far more widespread, occurring in most tissues except for the brain.

Strand asymmetries could potentially arise from RNA-specific edits or transcriptional errors. To test if these are *bona fide* DNA mutations, we examined exome data from matching blood samples and observed general agreement between the level of asymmetry per gene as measured by DNA vs RNA-seq (Fig. 3d).

In addition, we found blood samples to have the highest levels for C>T and T>C asymmetries. However, the directionality of these asymmetries suggests that samples with high biases may be driven by the two major types of RNA editing: A>I (represented in our data by T>C on the transcribed strand) and C>U (represented in our data by C>T on the non-transcribed strand) (Fig. 3a, right panel). Interestingly, estimating cell type abundances in each blood sample (see the “Methods” section) revealed that the abundances of two cell types, resting NK cells and CD8+ T cells, showed the strongest associations with the extent of both of these asymmetries (Additional file 1: Figure S7b,c). Consistent with this result, these same two cell types have been shown to have increased levels of both types of editing in stress conditions [30]. This suggests that some RNA editing sites may be present in our catalog of somatic mutations, though we did not observe an increased FDR for these two mutation types in the blood (Fig. 1d), suggesting that RNA editing has not substantially inflated our FDRs.

### Gene expression implicates pathways associated with somatic mutation load

**Fig. 4:**
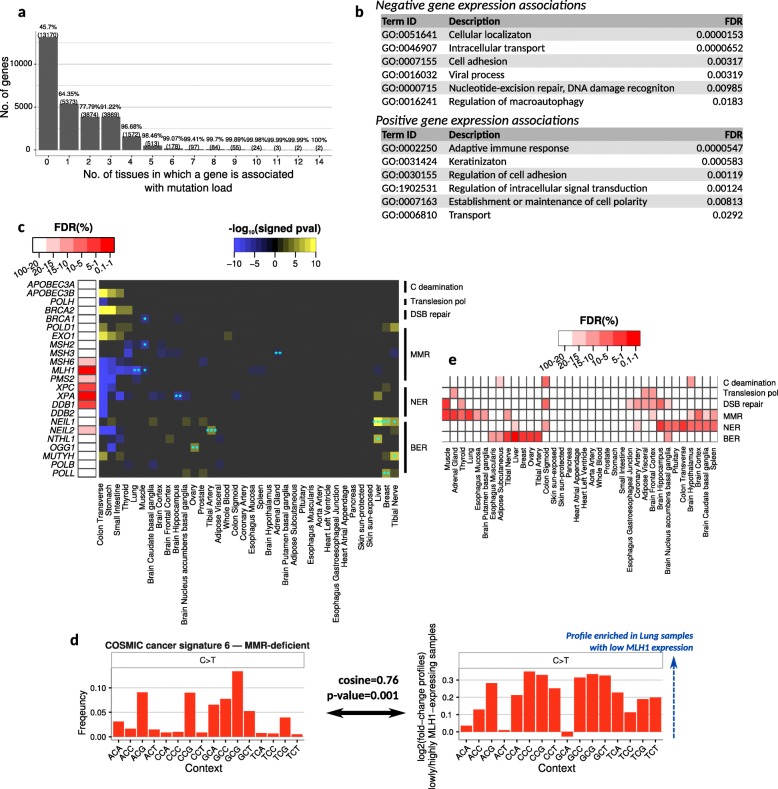
Mutation load is associated with the expression of genes and pathways. **a** Histogram of the number of tissues for which each gene was significantly associated with mutation load (Bonferroni-corrected *p* < 0.05). Associations were estimated for each tissue using linear models controlling for population structure and biological and technical cofactors (see the “Methods” section). **b** Genes whose expression was negatively (top) or positively (bottom) associated with C>T mutation load in multiple tissues are enriched in these representative GO categories (see the “Methods” section and Additional file 11: Table S8, Additional file 12: Tables S9). **c** Individual gene-tissue associations between C>T mutations and expression of genes involved in DNA repair or DNA mutagenesis (right panel). Blue asterisks denote significant associations using a permutation-based FDR strategy (see the “Methods” section; *FDR < 0.2, **FDR < 0.1, ***FDR < 0.05). Shown on the left panel are genes whose expression was associated with mutation load across all tissues more than expected by chance at the indicated FDR (see the “Methods” section). **d** COSMIC cancer signature 6 **(**linked to MMR deficiency; left) is significantly similar to profiles from lung samples expressing low levels of MLH1 (right). The plot in the left panel represents the frequency of C>T mutations at the indicated tri-nucleotide context for signature 6. The plot in the right panel is the log ratio of C>T mutation rates comparing the 20% of lung samples with the lowest MLH1 expression vs the 20% with the highest MLH1 expression. Cosine similarity is indicated for C>T mutation across contexts, and the *p* value represents the frequency of cosine similarity values that were greater than the original from permuted values—permutations were done by randomly selecting 2 groups of 20% of samples and calculating the fold-change mutation frequencies between them. **e** Group-level gene expression associations of the shown pathways and C>T mutations across tissues (see the “Methods” section). Heatmap columns in **c** and **e** are ordered based on a hierarchical clustering

To explore cellular factors accompanying an increase in somatic mutation load across non-disease tissues, we performed an unbiased tissue-level search for genes whose expression was associated—either positively or negatively—with exome-wide mutational load (see the “Methods” section). These enrichments may reflect a mixture of causal scenarios: gene expression impacting mutations or vice versa, or both driven by a third variable. Here we focused on the most abundant C>T mutations (Fig. 4; Additional file 1: Figure S8); other mutation types are described in the supplement (Additional file 1: Figures S9, S10).

While most genes were tested in the majority of tissues (Additional file 1: Figure S8a), the most significant associations were specific to only 1–3 tissues (Bonferroni-corrected *p* < 0.05; Fig. 4a, Additional file 1: Figure S8b). Among genes positively associated with mutation load in multiple tissues (see the “Methods” section), we observed enrichments for GO categories that included nucleotide excision repair, cellular transport, cell adhesion, and macroautophagy and categories including immune response, keratinization, and cell polarity for negative expression-mutation associations (Fig. 4b; Additional file 11: Tables S8, Additional file 12: Table S9). Consistent with these results, several of these same processes (e.g., cellular transport, autophagy, and cell adhesion) are known to be associated with mutation load in cancer or normal cells [31–33].

To further explore the contribution of different DNA repair pathways to mutagenesis, we focused on the associations between the expression of repair pathway members with mutation load. Specifically, we analyzed genes involved in double-strand break (DSB) repair, mismatch repair (MMR), nucleotide excision repair (NER), base excision repair (BER), the DNA deaminases *APOBEC3A/B*, and the translesion polymerase *POLH*. We found 14 associations at a < 20% FDR, 8 at < 10% FDR, and 2 at < 5% FDR (Fig. 4c blue asterisks; Additional file 1: Figure S8c-g) which are further described in Additional file 9: Note S2.

We then tested genes in these pathways for consistent associations with mutation load across tissues (see the “Methods” section; Fig. 4c, left panel; Additional file 1: Figure S10a-e). We observed significant hits from NER (*XPA*, FDR = 0.01; *XPC*, FDR = 0.07; *DDB1*, FDR = 0.07), MMR (*MLH1*, FDR = 0.05; *MSH6*, FDR = 0.16; *PMS2* FDR = 0.17), and BER (*NEIL2*, FDR = 0.15). The associations were generally in the expected direction, based on what is known about each gene (see Additional file 9: Note S3); for example, all associations of *MLH1* were negative, indicating lower expression associated with higher mutation load (Fig. 4c, Additional file 1: Figure S8e). If this association reflects a causal relationship, we hypothesized that tissue samples with low expression of *MLH1* might show a mutation profile suggestive of deficient mismatch repair. Supporting this hypothesis, we observed that in the lung—the tissue with the strongest association between *MLH1* expression and C>T mutation load—samples with low expression of *MLH1* had a significant resemblance to a cancer mutation signature that has been attributed to deficient MMR (Fig. 4d; similarity to COSMIC signature 6: cosine similarity = 0.76, *p* = 0.001 for C>T mutations, and cosine similarity = 0.28, *p* = 0.02 across all mutation types). *MLH1* silencing contributes to cancer development and mutagenesis [34], and our results suggest that natural variation of *MLH1* expression may affect mutagenesis in pre-cancerous tissues as well.

To further explore factors that may contribute to mutation load, we analyzed the expression of entire pathways or functionally related gene sets in each tissue (see the “Methods” section). MMR, BER, and NER are all strongly associated with mutation load in multiple tissues, but with very little overlap among them (Fig. 4e; Additional file 1: Figure S10f-j). As a result, these associations are primarily dominated by just one or two pathways per tissue.

In summary, most transcriptional signatures associated with mutation load are tissue-specific; however, genes associated with mutation load in several tissues are enriched in a number of pathways including DNA repair. These results paint a complex landscape where mutational load across non-disease tissues is associated with a variety of cellular functions.

### Cancer driver genes are enriched for mutations and under positive selection in non-disease tissues

**Fig. 5:**
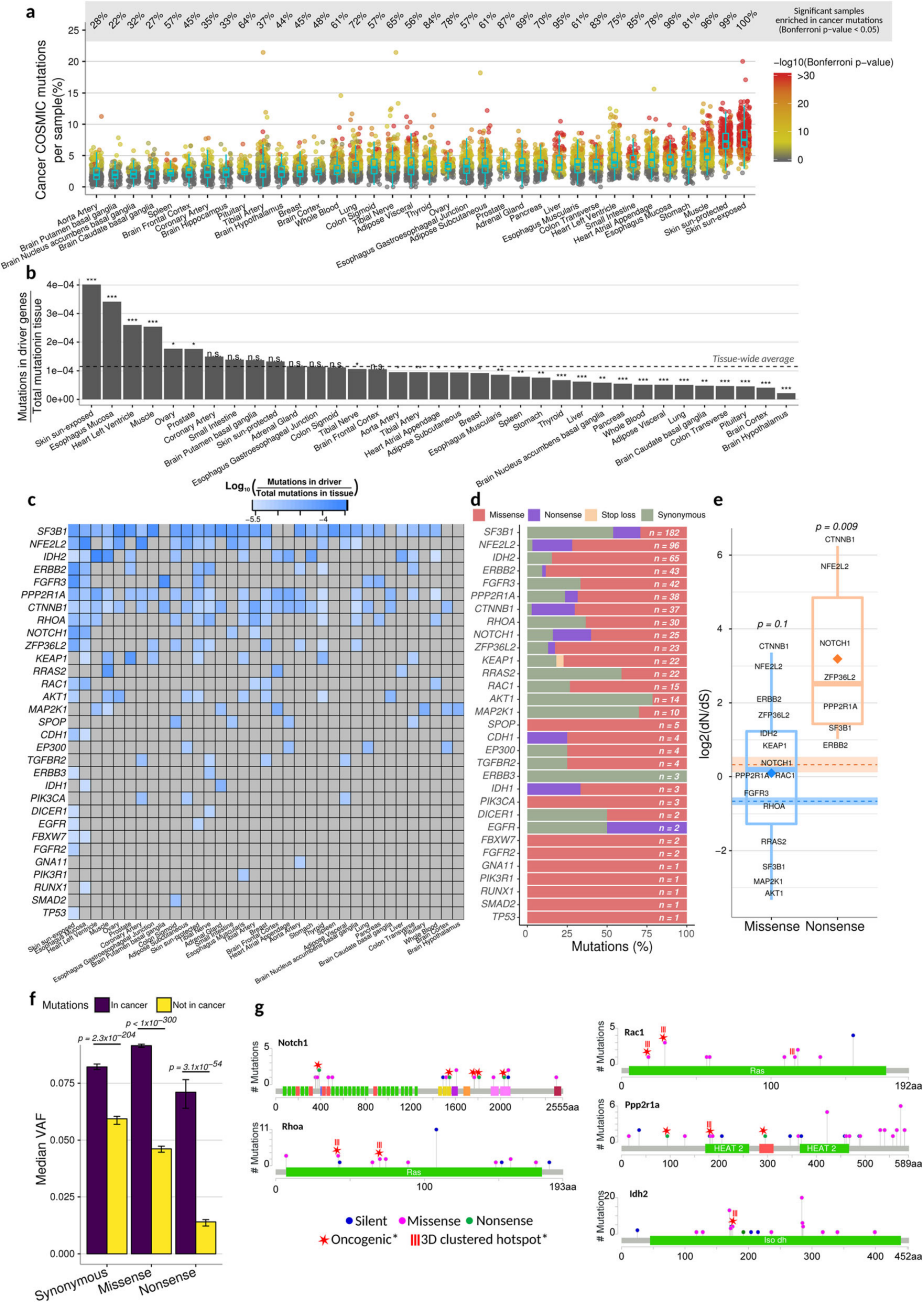
Cancer driver genes evolve under strong positive selection, and cancer mutations are enriched in healthy tissues. **a** Percentage of COSMIC cancer mutations observed per sample and grouped by tissue; *p* values for enrichment were calculated using a hypergeometric test accounting for sequencing coverage, total number of mutations per sample, total number of COSMIC mutations, and the three possible alternate alleles that any given reference allele can have (see the “Methods” section). *p* values are Bonferroni-corrected across all samples. **b** Relative mutation rates of a selected group of 53 genes known to carry cancer driver mutations [2] (only 31 of them had at least 1 mutation in this study); the tissue-wide average is indicated with the dotted line. Significant deviation from the tissue-wide average was calculated using the binomial distribution and the tissue-wide average mutation rate. Benjamini-Hochberg FDR: ***FDR < 0.001, **FDR < 0.01, *FDR < 0.05. **c** Individual mutation rates for each cancer driver gene across all tissues. **d** Percentage of mutations for each cancer driver gene stratified by impact to amino acid sequence; *n* is the total number of mutations observed in a gene. **e** dN/dS values for missense (blue) and nonsense mutations (orange) in cancer driver genes calculated using dndsloc [9, 36] (see the “Methods” section); averages per group are shown as rhomboids, and their respective genome-wide averages are shown as dashed lines along with their 95% confidence intervals after bootstrapping 10,000 times. *p* values indicate the probability of observing a higher average dN/dS from 10,000 equally sized randomly sampled groups of genes (see the “Methods” section). **f** Median variant allele frequency (VAF) for each mutation type based on their impact to the amino acid sequence and colored by their cancer status. Mutations “in cancer” (purple bars) are those that overlap with the COSMIC database in both base change and position, and mutations “not in cancer” (yellow bars) are those that overlap with COSMIC only in position but not in base change. Error bars represent the 95% confidence interval after bootstrapping 1000 times; *p* values are from two-sided Mann-Whitney tests. **g** Mutation maps of five cancer driver genes; oncogenic state was obtained from oncoKB [37], and clustered mutation annotations were obtained from the databases Cancer Hotspots [38] and 3D Hotspots of mutations occurring in close proximity at the protein level [39]

To investigate the extent to which cancer-associated mutations exist in a pre-cancerous state, we calculated the enrichment of all COSMIC [35] cancer point mutations in our mutation maps (see the “Methods” section). Many samples were highly enriched (hypergeometric Bonferroni-corrected *p* < 0.05), with some having more than 20% overlap with COSMIC mutations (Fig. 5a). In contrast, we observed almost no significant overlaps in a negative control (using a permuted set of mutations per-sample that conserves mutation frequencies and genomic regions from the original set of mutations; see the “Methods” section; Additional file 1: Figure S11a). The sun-exposed skin had the highest level of overlap (hypergeometric BH-FDR < 0.05), with 100% of samples having significant enrichments, followed by the sun-protected skin and skeletal muscle (Fig. 5a). We observed the lowest overlaps across the seven brain regions, aorta, and spleen.

Focusing our analysis on a panel of 53 known cancer driver genes [2] (Additional file 13: Table S10), we observed mutations in 31 of them (after excluding potential false-positive mutations; see the “Methods” section and Additional file 14: Table S11). Some tissues showed significantly greater or lower mutation rates across these genes (Fig. 5b). The sun-exposed skin was once again the most significant tissue, with several others at FDR < 0.05, such as the muscle and heart (Fig. 5b). Interestingly, *MAP2K1* and *RRAS2* are highly mutated in the muscle, and *IDH2* and *PPP2R1A* are highly mutated in both the heart and skeletal muscle (Fig. 5c). Conversely, several tissues—including most brain regions—had significantly lower mutation rates than average in these cancer drivers (Fig. 5b).

Observed mutations in cancer driver genes were then classified by their impact on protein sequences. We found that some genes were dominated by missense mutations, such as *IDH2*, while others showed an excess of synonymous mutations, such as *MAP2K1* (Fig. 5d).

To assess the selective pressures acting on these genes, we used a dN/dS approach, which assesses the ratio of non-synonymous to synonymous mutations while accounting for sequence composition and variable mutation rates across the genome [36] (see the “Methods” section). dN/dS values close to 1 represent little or no detectable selection, dN/dS > 1 suggests positive selection, and dN/dS < 1 suggests purifying selection. Our analysis showed that most of these cancer drivers are evolving under positive selection (Fig. 5e). Missense mutations had a mean dN/dS = 1.06 whereas nonsense mutations had a mean dN/dS = 9.14 (indicating over ninefold enrichment for nonsense mutations compared to the expectation based on synonymous mutations in the same genes); the latter is significantly more than expected from genome-wide values (permutation-based *p* = 0.1 and *p* = 0.009, respectively; Fig. 5e).

*NOTCH1* has been recently observed to evolve under positive selection in the non-cancerous skin [15] and esophagus [18, 19]. Consistent with this, we found the highest rates of *NOTCH1* mutations in these same two tissues (Fig. 5c), with strong signals of positive selection (*NOTCH1* missense dN/dS = 1.46, nonsense dN/dS = 14.04). We also observed *NOTCH1* mutations at slightly lower rates in the small intestine and tibial artery (Fig. 5c).

To further explore the effects of selection on cancer-associated mutations, we performed a VAF analysis (similar to Fig. 2d). Using the large catalog of COSMIC cancer point mutations (as in Fig. 5a), we created two subsets of our somatic mutation list: those that have been observed in cancer samples and those at precisely the same location as a known cancer mutation, but with a different base change (which act as well-matched negative controls). We then separated each of these two subsets into three mutation types: synonymous, missense, and nonsense. In all three cases, the VAFs of the cancer-associated mutations were significantly higher than the matched non-cancer-associated ones (Fig. 5f), suggesting that cancer-associated mutations often increase cellular proliferation rates (and thus VAFs). Using the synonymous non-cancer mutations as representative of neutrality (similar to the logic of dN/dS), we found that all three cancer-associated mutation types had higher VAF than these neutral proxies (Fig. 5f), suggesting the action of positive (and not just weaker negative) selection. Interestingly, the ratio of cancer/non-cancer mutation VAFs was lowest for synonymous (1.4-fold), intermediate for missense (2.0-fold), and highest for nonsense (5.2-fold), suggesting stronger positive selection for more extreme mutations. In addition, cancer-associated mutations were found in more individuals compared to other mutations, particularly in non-brain tissues (median of 1.7-fold more individuals for non-brain mutations, and 1.2-fold for the brain). These results are consistent with the dN/dS analysis in Fig. 5e, which also showed the strongest positive selection on nonsense mutations in cancer driver genes, but the VAF analysis has substantially more power due to the greater number of mutations analyzed.

To explore a subset of cancer driver mutations in more detail, we identified specific mutations in our catalog that have been manually curated as oncogenic or likely oncogenic from OncoKB [37] (Additional file 15: Table S12). For instance, all nonsense mutations that we observed in *NOTCH1* have been labeled as oncogenic (Fig. 5g). We found many other oncogenic mutations as well; for example, *RHOA* and *RAC1* had oncogenic mutations in their Ras domains (Fig. 5g).

Together, these results show that cancer mutations are enriched in non-disease tissues and not only do they accumulate in driver genes, but the majority of these genes are evolving under positive selection, suggesting that these mutations increase cellular proliferation well before any cancer is observed.

## Discussion

We developed a method to detect rare somatic mutations from RNA-seq data and applied it to over 7500 tissue samples (Fig. 1). To our knowledge, this is the largest map to date of somatic mutations in non-cancerous tissues.

It has been proposed that somatic mutations contribute to aging and organ deterioration [40, 41]; consistently, we observed a positive correlation between age and mutation burden in most tissues. Interestingly, several brain regions are among the tissues exhibiting stronger age correlation, and somatic mutations have been shown to have a role in neurodegeneration [23].

We observed largely tissue-specific behaviors and some pervasive observations shared across tissues, providing genome-wide evidence in humans consistent with earlier gene-level findings in mouse models [42]. Mutation profiles are defined by their tissue-of-origin, mutation maps are delineated by tissue-specific chromatin organization, and transcriptional signatures associated with mutation load are highly tissue-specific. These results suggest that different cell types are subjected to different evolutionary paths that could be dependent on environmental or developmental differences. For example, while most samples exhibit tissue-specific mutation profiles, some others like transverse colon and the small intestine have similar profiles. Additionally, we observed that genes whose expression is associated with mutation load in several tissues are enriched in DNA repair, autophagy, immune response, cellular transport, cell adhesion, and viral processes, and while these functions have been implicated in mutagenesis in cancer [31–33], our results highlight how expression variation of these genes associates with mutational variation in healthy tissues.

Cancer mutations are enriched across many organs. Muscle and heart tissue are particularly interesting because they have lower-than-expected mutation rates, but those mutations are highly enriched for cancer mutations and had high mutation rates in cancer drivers. Sarcomas are tumors originated from soft tissues—including muscle—that are relatively uncommon and have a low density of point mutations but high copy number variation [43]. Our results show that low mutation rates are also observed in these soft tissues, but compared to tumors, cancer mutations are frequent. The functional implications of this observation will need to be explored in further detail.

Positive selection of driver genes has been recently observed in healthy tissues [15, 18]. Accordingly, we confirmed that *NOTCH1* is under positive selection in the skin and esophagus. We found other genes positively selected both broadly (e.g., *IDH2*, *CTNNB1*, *NFE2L2*) and with more tissue-specific patterns (e.g., *KEAP1* in the prostate, thyroid, and muscle; *RAC1* in the skin, esophagus, and breast), which will be important subjects for future studies.

In general, mutations previously observed in cancer studies were found in high abundance across many healthy tissues, and our VAF analysis showed signatures of positive selection acting on them. In contrast, when looking at all mutations and in particular those not previously seen in cancer, we found that missense and nonsense mutations are generally under negative selection. Our results reconcile recent studies reporting prevalent positive [9] or negative [11, 44] selection in somatic evolution, as we find evidence of both co-existing in different sets of mutations.

After this manuscript was submitted for publication, another study was published that also called mutations using GTEx samples [45]. Reassuringly, their results are largely consistent with ours, including the higher mutation rate in female breast tissue (vs male) and in the sun-exposed skin (vs the non-sun-exposed skin) of Caucasians but not African-Americans, as well as the excess of nonsense mutations in *NOTCH1*. However, a major difference between the two studies is in the number and confidence of mutations called. Specifically, Yizhak et al. called 8870 somatic mutations in 6707 RNA-seq samples; this is 1.3 mutations per sample, which is actually smaller than their expected 2–4 false positives per sample, suggesting that most of their calls are false positives. This is supported by their validation of 5/28 tested variants (82% FDR) in GTEx samples and 13/86 variants (85% FDR) in TCGA cancer samples. In contrast, we called over 280,000 mutations with a 34% FDR, based on thousands of validated variants in exome data. Thus, our method called ~ 115 times as many true positive mutations, at substantially higher confidence (Additional file 1: Figure S12a). Additionally, 632 (7%) of their mutation calls were flagged by one or more of our filters in the corresponding tissues (Additional file 1: Figure S12b). As a result of our increased power, we were able to connect somatic mutations with tissue-specific chromatin and gene expression levels (Fig. 2g, Fig. 4), detect both negative and positive selection acting on different subsets of mutations (Fig. 2d, Fig. 5e, f), and discover widespread strand asymmetry (Fig. 3). One notable difference is that Yizhak et al. detected *TP53* and *PIK3CA* as the genes with the most hotspot mutations genome-wide, whereas these were not outliers in our analysis (Fig. 5d). However, it should be noted that these two genes were also the only two genes that Yizhak et al. excluded from their “panel of normals” filter when calling mutations, which of course inflates their number of mutations, making this result difficult to interpret.

## Conclusions

Our findings paint a complex landscape of somatic mutation across the human body, highlighting their tissue-specific distributions and functional associations. The prevalence of cancer mutations and positive selection of cancer driver genes in non-diseased tissues suggests the possibility of a poised pre-cancerous state, which could also contribute to aging. Finally, our method for inferring somatic mutations from RNA-seq data may help accelerate the study of somatic evolution and its role in aging and disease.

## Methods

### Raw RNA-seq retrieval and processing

We downloaded raw RNA-sequencing reads from the dbGAP GTEx [46] project version 7 (phs000424.v7.p2). We included 36 tissues with a total of 7584 samples (Additional file 4: Table S2) after applying all filters in the mutation calling pipeline (see below). We also processed DNA exome sequencing reads from 105 whole-blood samples that had a matched RNA-seq sample.

Reads were mapped to the human genome Hg19 (NCBI build GRCh37.p13) using STAR [47] with the following parameters: requiring uniquely mapping reads (--outFilterMultimapNmax 1), clipping 6 bases in the 5′ end of reads (--clip5pNbases 6), and keeping reads with 10 or fewer mismatches and less than 10% mismatches of the read length that effectively mapped to genome (--outFilterMismatchNmax 10, --outFilterMismatchNoverLmax 0.1). To avoid germline variant contamination during the somatic mutation calling phase, all SNPs from dbSNP [48] v142 were masked to Ns and ignored in downstream processing (https://ftp.ncbi.nlm.nih.gov/snp/organisms/human_9606_b142_GRCh37p13/BED).

After mapping, we removed duplicate reads to avoid biases arising from PCR duplicates during library preparation using a custom python script.

### DNA somatic mutation calling from RNA sequencing

Identifying DNA variants from RNA-seq requires filtering out many sources of false positives. These include sequencing errors, RNA editing events, mapping errors around splice junctions, germline variants, and other sequencing/mapping biases. To address these issues, we developed a custom mutation calling pipeline that borrows ideas from classical DNA-based variant calling coupled with extra filters to increase the fidelity of the mutation calls from RNA-seq.

After mapping the raw RNA-seq reads, the pipeline consists of three main parts: (1) identifying positions with two base calls, (2) removing germline variants, and (3) filtering out other potentially spurious variants.

#### Identifying positions with two base calls

After mapping, bam files were scanned to identify genomic positions that were covered by reads having exactly two different base calls. Given the intrinsic sequencing error rate, we only considered positions with high coverage and high sequencing quality for this step. Stringent cutoffs were set for coverage (*c* ≥ 40 reads) and sequencing quality (*q*_s_ ≥ 30 in Phred scale); this is in comparison twice what some DNA-based calling algorithms have used [21]. Finally, only positions in which the minor allele was supported by at least 6 reads were considered (*n* ≥ 6). In combination, these parameters define a theoretical distribution of the probability of observing a mutation due to sequencing errors, which is small for a wide range of sequence coverage levels (Additional file 1: Figure S1a). In addition, we included a filter to only consider variants with a probability of sequencing error *p* < 0.0001 (see filters below). These strict cutoffs ensure a low probability of observing sequencing errors even in highly covered genes; nonetheless, we still apply further filters to account for sequencing errors (see below).

#### Identifying and eliminating germline variants

Common and rare germline variants have to be excluded for the proper identification of somatic mutations. To eliminate common variants, a strict filter was applied by using a human genome masked with Ns for positions known to have common variants from dbSNP v142 (see above).

To eliminate all other germline variants, including rare ones, we utilized the low confidence germline variants called by GTEx. These calls were made by GTEx using GATK’s HaplotypeCaller v3.4 on whole-genome sequencing data at 30x coverage from whole-blood samples. We specifically used the vcf file *GTEx_Analysis_2016-01-15_v7_WholeGenomeSeq_652Ind_GATK_HaplotypeCaller.vcf* which contains all germline variants before filtering for MAF and low-quality variants. Our goal was to exclude as many germline variants as possible, so we reasoned that using all germline variants called by GTEx—including the low-quality ones—was the safest option to minimize germline mutation contamination in our somatic mutation calls. While these variants were called in whole-blood samples, germline variants should be present in all tissues of an individual and as such these variants were excluded in a per-individual basis across all tissues of that individual. Only sites that had heterozygous or homozygous variants for the alternate allele were excluded from the mutation calls of the given individual.

#### Filtering out artifacts

A total of 13 filters were applied to exclude a variety of artifacts:

1. Blacklisted regions. We excluded sex chromosomes, unfinished chromosomal scaffolds, the mitochondrial genome, and the HLA locus in chromosome 6 which is known to contain a high density of germline variants [49] making accurate mapping challenging and is hence a potential source of false-positive calls.
2. RNA edits. The most prevalent type of RNA editing in humans is Adenine-to-Inosine (A>I) which is observed as an A>G/T>C substitution in sequencing data. The Rigorously Annotated Database of A-to-I RNA editing (RADAR) [50] has extensively curated RNA-edit events including calls identified using the GTEx data [51]. We also obtained RNA-edit information from the Database of RNA editing (DARNED) that includes further edit types curated from published studies [52]. We excluded all positions described in RADAR v2 (http://lilab.stanford.edu/GokulR/database/Human_AG_all_hg19_v2.txt) and in DARNED (https://darned.ucc.ie/static/downloads/hg19.txt) and observed an average decrease of 10% mutations per sample in our RNA-sequencing calls but not in our DNA-sequencing calls from GTEx (Additional file 1: Figure S2a), indicating that we are indeed eliminating real RNA-edit events that would otherwise be false-positive mutation calls.
3. Splice junction artifacts. Splice junctions are difficult to resolve during mapping because a gap has to be introduced in reads spanning a splice junction to map it to the corresponding exons in the genome. We observed that the mutation rate was higher close to annotated exon ends and it stabilized at ~ 7 bp away from the exon end across all tissues (Genecode v19 genes v7 annotation file) (Additional file 1: Figure S1b). Most of these are likely mapping artifacts, and we therefore excluded all mutations located less than 7 bp away from an annotated exon end.
4. Sequencing errors. While sequencing errors are unlikely to be found given the parameters established in the first part of the mutation calling pipeline (see above), we additionally filtered out mutations that had a probability of being sequencing errors greater than 0.01%. This probability was calculated using the upper tail integral of the binomial distribution where the number of successes is the number of reads supporting the alternate allele, the number of events is the coverage in that position, and the probability of success is the conservative assumption of *p* = 0.001 which equals our cutoff of phred score 30 during the first part of the pipeline (see above). This is extremely conservative because it does not incorporate the probability of observing the exact same base call across all reads supporting the alternate allele.
5. Read position bias. To eliminate any systematic bias of a mutation being consistently called around the same position along reads supporting it versus the rest of the reads, we excluded mutations that had a *p* value less than 0.05 when applying a Mann-Whitney *U* test of the positions in the read supporting the alternate allele vs the positions supporting the reference allele. For these tests, we used BCFtools [53] *mpileup.*
6. Mapping quality bias. We excluded mutations that had a *p* value less than 0.05 when applying a Mann-Whitney *U* test comparing mapping quality scores of the base calls supporting the alternate allele vs the mapping quality scores of reads supporting the reference allele. For these tests, we used BCFtools [53] *mpileup.*
7. Sequence quality bias. We excluded mutations with a *p* value less than 0.05 when applying a Mann-Whitney *U* test comparing sequencing quality scores of base calls supporting the alternate allele vs the scores of base calls supporting the reference allele. For these tests, we used BCFtools [53] *mpileup.*
8. Strand bias. We excluded mutations with a *p* value less than 0.05 when applying a Mann-Whitney *U* test comparing strand bias of bases supporting the reference and alternate allele (i.e., cases where mutations were only observed on one strand were excluded; this is not related to the strand asymmetry we observed for some mutation types). For these tests, we used the “Mann-Whitney *U* test of Mapping Quality vs Strand Bias” of BCFtools [53] *mpileup.*
9. Variant distance bias. We excluded variants that showed a high or low mean pairwise distance between the alternate allele positions in the reads supporting it. Similar to the *read position bias* filter, this ensures that we filter mutations that are consistently observed around the same region of all the reads that support it. We used a cutoff of *p* < 0.05 for a two-tail distribution of simulated mean pairwise distances from the BCFtools [53] implementation.
10. RNA-specific allele frequency bias. We observed an enrichment of variants having a variant allele frequency (VAF) greater than 0.9 only in mutation calls from RNA-sequencing but not from the matched DNA-sequencing samples. This RNA-specific bias could be due to several factors, including allele-specific expression leading to enrichment of the alternate allele, RNA editing, or systematic artifacts during RNA extraction, library construction, and sequencing. We took a conservative approach and excluded all mutations that had a VAF greater than 0.7.
11. Tissue-specific mutation effects. To eliminate false positives arising by systematic artifacts of unknown origin, we first looked for recurrent mutations observed in many samples of a given tissue. While these mutations could be real and have a biological impact, they may also reflect a shared systematic artifact that is producing the same exact mutation across several samples of the same tissue. We decided to take a conservative approach and eliminated all mutations that were called in at least 40% of the samples in one tissue. Even though we labeled these mutations on a per-tissue basis, once identified, we removed them from any sample in any tissue that had them.
12. Overall systematic mutation bias. We further eliminated mutations that were present in at least 4% of all samples. Similarly, as in the previous step, these mutations are more likely to have originated from a systematic artifact.
13. Hyper-mutated samples. We excluded samples that had an excess of mutations compared to what it was expected from sequencing depth and biological factors. To do so, we looked at the residuals after applying a linear regression on mutation numbers using as features sequencing depth, age, sex, and BMI. We observed 48 samples that had residual values greater than 1500 (i.e., they had > 1500 more mutations than expected by other factors) (Additional file 1: Figure S1 g) and excluded them from further analysis, leaving 7584 remaining. We did not observe any hypo-mutated samples having similar residual values in the opposite direction.

## Method validation

To validate the precision of our DNA somatic mutation calls from RNA-seq, we sought to compare those calls to DNA sequencing from the exome. The GTEx Consortium performed DNA exome capture followed by sequencing in blood samples with a median coverage of 80x.

From the GTEx dbGaP repository (phs000424.v7.p2), we downloaded raw DNA exome sequencing runs from 105 randomly chosen donors. We mapped the reads and called mutations identically to our RNA-seq samples. Throughout the study, we used the mutation calls from exome to validate certain results when appropriate.

To validate the overall mutation calling pipeline, we matched RNA-seq to exome DNA-seq samples of the same individuals. Then, we asked what percentage of the mutation calls from RNA-seq had reads supporting the alternate allele in the matched exome DNA-seq. To account for coverage differences between RNA-seq and DNA-seq, we focused this analysis only on mutation calls for which we had reasonable power of detection to validate mutations in DNA-seq data. This is especially relevant because in highly expressed genes, sequencing coverage can be > 1000x in RNA-seq; therefore, we could detect mutations with VAF as low as 0.006 for a 1000x-covered position (given our minimum alternate allele 6-read cutoff described above). In comparison, in exome DNA-seq given the 80x median coverage, we expect to see only 0.48 reads supporting the alternate allele of a variant with VAF = 0.006. We therefore devised a way to account for this coverage difference between RNA-seq and DNA-seq, and only compared positions where we had power of detection in both experiments. We first made the following calculation:

$$ {r}_i={C}_{i,\mathrm{DNA}-\mathrm{seq}}\left(\frac{A_{i,\mathrm{RNA}-\mathrm{seq}}}{C_{i,\mathrm{RNA}-\mathrm{seq}}}\right) $$

where *C*_*i*_ and *A*_*i*_ are total and alternate read counts for a mutation in position *i* and *r*_*i*_ effectively represents the number of expected reads to support the alternate allele in DNA-seq at position *i* given the VAF observed in RNA-seq. As shown in Additional file 1: Figure S1f, we took a conservative approach and only compared genomic positions where *r* ≥ 8, to ensure sufficient power of validation. Since this method will inherently select mutations in a way that shifts the VAF distribution towards higher values, we have estimated a VAF-corrected FDR by binning all validation variants by VAF (as in Additional file 1: Figure S1d) and then calculating an average FDR weighted by the fraction of all mutation calls present in each bin. For example, if 20% of mutations had a VAF-matched FDR of 25% and 80% of mutations had a VAF-matched FDR of 30%, then the overall FDR would be 29%. Applying this approach, our VAF-corrected FDR was 34%.

For all positions selected, we then calculated the percentage of mutations observed in an RNA-seq sample that had at least one read supporting the variant allele in the exome DNA-seq, effectively resulting in a false-discovery rate. When assigning a random alternate allele (not including the reference allele among the choices) to the mutated positions in RNA-seq, we did not observe support for > 98% of the position-permuted mutations, validating this FDR calculation (Additional file 1: Figure S1c).

### Discovery of mutation associations with biological and non-biological factors

We first sought to identify and subtract the effects of non-biological factors influencing the number of mutations observed per sample. To this end, we grouped samples by tissue and performed a linear regression on the mutation numbers using non-biological factors as explanatory variables, which included sequencing depth, transcriptome Shannon diversity, time spent in the PAXgene fixative (SMTSPAX from GTEx sample attributes), total ischemic time (SMTSISCH from GTEx sample attributes), and RNA integrity number (SMRIN from GTEx sample attributes). We assessed the significance of association between mutation number and any factor by the *p* value of each coefficient in a multiple regression (Additional file 6: Table S4).

To discover biological factors associated with mutation numbers, we took the residuals of the previous linear regression (constructed with non-biological explanatory variables) and performed another regression using biological factors as explanatory variables. Biological factors included biological sex, age, BMI, and the first three genotype principal components constructed from the ~ 10^7^ SNPs with MAF ≥ 0.1 available from GTEx. Significance was assessed by the *p* value of each coefficient in a multiple regression (Additional file 6: Table S4). Additionally, Spearman correlations were independently performed between each biological factor and the residuals from the linear regression between mutation number and non-biological factors.

### Analysis of sample mutation profile similarity (tSNE)

To assess the mutation profile similarity across samples and to evaluate their tissue specificity, we performed clustering analysis on mutation profiles using tSNE followed by silhouette scoring.

tSNE was performed on a matrix of *m* rows and *n* columns, where *m* are samples and *n* are mutation type counts. We used a 1536-type mutation profile including two base-pairs upstream and downstream of the mutation site (pentanucleotide profile).

The content of the background pentanucleotide sequences can be influenced by sample-specific gene expression, which in turns shapes the probability of observing mutations across different pentanucleotide sequences, thus obscuring the underlying *de facto* mutation profile. To account for these background sequence differences driven by expression differences, we used a normalized mutation count. The normalized mutation count (*mut*_*i*_) was obtained as follows:

$$ {mut}_i=\frac{c_i}{\sum \limits_g^{genes}{s}_{g,i}{e}_g} $$

where *c*_*i*_ is the count of the mutation type *i*, *s*_*g,i*_ is the number of occurrences of the *i* sequence context in gene *g*, *e*_*g*_ is the expression in TMP of gene *g*, and *genes* are all genes with TPM ≥ 1 in the given sample. This calculation was performed separately for every sample. We used the tSNE implementation in R (Rtsne) with parameters dims = 2, max_iter = 500, perplexity = 30, pca = TRUE, theta = 0.5.

To quantify the clustering among different groupings in the tSNE two-dimensional space, we calculated a silhouette score (SS) for each group defined in Fig. 2f by first obtaining a silhouette score (*s*_*i*_) for each sample in each group:

$$ {s}_i=\frac{b_i-{a}_i}{\max \left({a}_i,{b}_i\right)} $$

where *a*_*i*_ is the average distance of sample *i* to all other samples inside the group and *b*_*i*_ is the average distance of sample *i* to samples outside the group. We then calculated the average score *s* of all samples in a given group and the 95% confidence intervals based on bootstrapping 10,000 times.

We performed the individual-based grouping by calculating silhouette scores for all tissues from 20 randomly selected individuals and then averaging them. The random expectation was calculated by permuting the tissue labels across samples 10 times, repeating the SS calculation across tissues and then averaging all SSs.

### Cell type decomposition for blood and lung samples

We used CIBERSORT [54] to identify the cell type composition of each whole-blood sample in the GTEx data. Briefly, CIBERSORT applies a support vector regression on a gene expression profile(s) using reference gene expression signatures from different cell types, and then retrieves the cell type composition from the signatures in the original expression profile(s). We used the online portal (https://cibersort.stanford.edu/) and the default LM22 expression signatures composed of the most prevalent immune cell types. CIBERSORT was run on the blood gene expression profiles with default parameters: 100 permutations and “absolute” mode.

### Gene expression associations

To avoid population-based effects for all expression association analyses in this study, we only used self-reported Caucasian people.

For each tissue, we used individual gene expression to model mutation counts in a linear regression as follows:

$$ {p}_i={\beta}_0+\sum \limits_n{\beta}_n{\mathit{\operatorname{cov}}}_{n,i}+\gamma {x}_i $$

where *x* is the expression of a given gene in TPM, *cov* is a covariate variable, and there are *n* covariates. To estimate the effects of a SNP on the phenotype, γ is calculated for each gene in the genome that has a TPM > 1 in at least 20% of the samples. Significance is measured based on the *p* value of a non-zero *t* test performed on γ. *p* values are adjusted using Bonferroni correction.

When *p*_*i*_ is the vector of mutation loads, we defined it as the residuals after regressing non-biological factors as described previously (see above in the “Discovery of mutation associations with biological and non-biological factors” section). The covariates included in this model are the first 3 PCs of the whole-genome genotypes, age, sex, and BMI.

In all cases, both *p*_i_ and γ were normalized by converting the values into quantiles and mapping them to the corresponding values of the standard normal distribution quantiles, following standard GTEx practices [46].

### Gene Ontology analysis

For gene expression associations with mutation load, we assessed enrichment in GO biological processes using GOrilla [55]. We used as input a ranked list of genes based on the number of tissues they were significant in (and only including genes that were tested in more than the median number of tissues all genes were tested in, *p* < 0.05 after Bonferroni correction, see above in the “Gene expression associations” section) and breaking ties by significance. We then used REVIGO [56] to obtain non-redundant categories.

### Expression associations with genomic instability genes

We selected a panel of genes known to be involved in different pathways of DNA repair or translesion replication (Fig. 4) and performed association analyses between their expression and the mutational load for all different mutation types on a per-tissue basis. We followed the same linear regression strategy as described above in the “Gene expression associations.” We devised customized strategies to calculate FDRs for individual gene-tissue associations, gene enrichment across all tissues, and pathway (gene group) associations in each tissue.

For individual gene-tissue associations, we calculated an FDR based on the distribution of *p* values of the linear regressions from the tests of all genes in the genome (see above for additional filters). Then, for each gene of interest, we calculated the FDR as the percentage of genes from all tests that had an equal or lower *p* value.

To obtain an FDR of the enrichment for different pathways across tissues, for each pathway of interest, we obtained the *p* values from the individual linear regressions of each gene. We then created 10,000 groups with randomly selected genes with the same size as the pathway of interest and within the tissue of interest and performed linear regressions with mutation load (as described above in the “Gene expression associations” section). Finally, for each *p* value in the original group, we calculated the FDR by dividing the percentage of *p* values of equal or lower value in all permuted regressions by the percentage of *p* values of equal or lower value in the original regressions. For example, if 0.1% of permuted regressions reached *p* < 0.001, compared to 1% of unpermuted regressions, the FDR at *p* < 0.001 would be 0.1/1 = 10%.

Lastly, to calculate FDR for the enrichment of individual genes across all tissues, we used a similar strategy as the FDR calculation for gene pathways described in the previous paragraph. We first obtained the *p* values of individual linear regression of a given gene across all tissues. We then created a permuted set of *p* values from the regressions of one gene from each of the 10,000 permuted groups in all tissues. Finally, we calculated the FDR of the *p* values in the original group (one across all tissues) by dividing the percentage of *p* values of equal or lower *p* value in all permuted regressions by the percentage of *p* values of equal or lower value in the original regressions.

### Chromatin analysis

To address the influence of chromatin on somatic mutations, we first manually mapped tissues from GTEx to tissues of the Roadmap Epigenomics Project [27]. We were able to do such mapping for 18 tissues (Additional file 10: Table S7). For each tissue, on a per-exon basis, we calculated both mutation rates and signal for H3K36me3, H3K4me1, H3K4me3, H3K27me3, and H3K9me3.

To account for sequencing depth (expression) of an exon when calculating mutation rates, we assigned exons to 100 bins based on their sequencing depth, where each bin contains 1% of exons. We then calculated the median number of mutations observed in each bin, and finally, the mutation rate per exon was obtained by subtracting expected number of mutations (the median for all exons in that bin) from the observed number for that exon.

Chromatin signal for each exon was obtained from Roadmap ChIP-seq data. We calculated the average base-pair ratios of IP/input obtained from the Roadmap bigwig files.

Significance of the association between each histone modification and mutation rate was assessed by applying a linear regression on the mutation rate using all histone modifications as features and assessing the significance (*p* value) of each coefficient (histone modification).

Individual effect sizes (*r*) for each histone modification were obtained as follows: for each histone modification, a linear regression was performed using the rest of the histone modifications as features. The residuals of those regressions were then used in a separate linear regression with mutation rate as the response variable, from which the variance explained (*r*^2^) was then obtained.

### Mutation enrichment in COSMIC cancer mutations

We downloaded the entire set of cancer mutations from COSMIC [35] v86, and we further filtered them to only keep single nucleotide variants without indels. For each sample, we calculated the percentage (overlap) of their mutations that are present in COSMIC mutations, and we calculated the significance of the overlap using the integral of the upper tail from a hypergeometric distribution, that is *p*(*X* > *k*), where *X* follows a hypergeometric distribution with parameters *K* (number of mutations in sample), *n* (number of COSMIC mutations whose positions were covered by ≥ 40 reads in the RNA-seq sample), *N* × 3 (the total number of base pairs covered by ≥ 40 reads in the RNA-seq sample multiplied by the three possible alternate alleles that any reference can have), and *k* (the overlap of mutations, *K* and *n*). As a control, for each sample, this calculation was repeated using a permuted set of mutations. This set was constructed by randomly selecting genomic positions that were covered by ≥ 40 reads in the sample and then mutations were simulated in those positions. We preserved the number of reference alleles and their corresponding alternate alleles from the original mutations in this permuted set.

### dN/dS analysis

To calculate dN/dS ratios, we applied a previously described method [36] that uses a Poisson distribution to model the number of mutations with different impacts (i.e., synonymous vs non-synonymous). Briefly, the Poisson distribution is based on the relative content of a mutation type (e.g., C>T) across all types, the total content of that mutation type, and the density of mutations per site. For non-synonymous mutations, an extra parameter represents the effect of selection (dN/dS), and maximum-likelihood estimates are calculated by Poisson regression for all parameters. This framework accounts for different substitution rates across different genes as well as sequence composition. We used dndsloc, which is the implementation of this method in R [9] (https://github.com/im3sanger/dndscv).

### Mutation analyses on cancer driver genes

We downloaded the list of genes known to contain at least one cancer driver mutation, based on the latest TCGA publication on cancer driver genes [2] (Additional file 13: Table S10). Since we did more in depth analysis in this set of genes, we further removed potential false-positive mutation calls that could bias gene-level analysis but are not necessarily an issue for genome-wide analysis. For some of these genes, we observed a high number of total mutations but low number of unique mutations; in other words, some mutations accounted for most of the unique mutated sites, which may be artifactual [9]. To flag these events, for each mutation, we calculated a metric *t* as the ratio of the counts of that mutation divided by the unique number of mutations found in the gene-of-origin. Mutations with high *t* values account for most of the unique mutated sites in their gene-of-origin, leading to a low diversity in mutations of a given gene. These mutations may be artifacts and can bias dN/dS ratios [9]. The distribution of *t* values for mutations in this set of genes was bimodal (Additional file 9: Note S11b), and we therefore excluded mutations with *r >* 3.5.

To assess the mutation load on these genes and their significance, for each tissue, we calculated the mutation rate of these genes and then calculated whether this mutation rate was higher or lower than expected from the overall mutation rate of these genes across all tissues (Fig. 5b). To do so, we calculated the overall mutation rate in cancer driver genes across all tissues (*k*) and then for each tissue we calculated the probability of the number of observed mutations (*n)* in these genes using the binomial distribution [*X ~ binom(n,k)*]. For tissues showing more mutations than expected, we used the integral of the right tail of the binomial distribution to calculate the probability of observing n mutations, and conversely, for tissues showing fewer mutations, we used the integral of the left tail of the distribution. FDR was calculated using Benjamini-Hochberg method on the *p* values.

We annotated the oncogenic status for the mutations in these driver genes using the oncokb [37] tool *MafAnnotator.py*.

## Supplementary information

**Additional file 1:** **Figure S1.** Statistics associated to a method for calling DNA mutations from RNA-seq data. **Figure S2.** Calling of somatic DNA-mutations in the GTEx cohort. **Figure S3.** Mutation load across different mutation types in non-disease human tissues. **Figure S4.** Phenotypic associations and properties of mutation load in the human body. **Figure S5.** Number of stem cell divisions correlates weakly with mutation load in human tissues. **Figure S6.** Mutation profiles cluster by tissue. **Figure S7.** Inter-tissue mutational strand asymmetry correlations and cell type associations. **Figure S8.** Gene expression associations with C>T mutation load. **Figure S9.** Gene expression associations with overall mutation load. **Figure S10.** Mutation load associations with expression of genes involved in DNA repair or DNA mutagenesis. **Figure S11.** Negative controls and filters for cancer mutation enrichment in non-disease human tissues. **Figure S12.** Comparison of mutation calls to those from Yizhak et al.

**Additional file 2: **Legends for **Tables S1–S13. (PDF 40 kb)**

**Additional file 3: Table S1.** Average percentage elimination of putative mutation calls by per-sample false-positive filters across all tissues. (TSV 5 kb)

**Additional file 4: Table S2.** Total number of samples per tissue included for the final set of mutation calls. (TSV 784 bytes)

**Additional file 5 : Table S3.** List of all somatic mutations identified in this study. (TSV, 187 MB). (TSV 182623 kb)

**Additional file 6: Table S4.** *P*-values (−log10[p-value]) for the coefficients of each feature used in a linear regression on the total number of mutations per tissue. (TSV 6 kb)

**Additional file 7: Table S5.** Average percentage of each mutation type across samples of the given tissue. (TSV 3 kb)

**Additional file 8: Table S6.** Significant associations between biological sex and mutation load across tissues and mutation types. (TSV 854 bytes)

**Additional file 9: Notes S1**–**S3.**

**Additional file 10: Table S7.** Tissue correspondence between the GTEx [46] and Roadmap Epigenomics projects [27]. (TSV 1 kb)

**Additional file 11: Table S8** Significant GO enrichments for genes whose expression is significantly and negatively associated with C > T mutation load across several tissues. (TSV 2 kb)

**Additional file 12: Table S9.** Significant GO enrichments for genes whose expression is significantly and positively associated with C > T mutation load across several tissues. (TSV 1 kb)

**Additional file 13: Table S10.** List of cancer driver genes used in this study [2]. (TSV 312 bytes)

**Additional file 14: Table S11.** List of mutations in cancer driver genes after further elimination of potential false positives. (TSV 97 kb)

**Additional file 15: Table S12.** List of mutations in cancer driver genes annotated in Oncokb [37]. (TSV 4 kb)

**Additional file 16.** Review history

## Data Availability

• All code and software used in this study is available through github (https://github.com/pablo-gar/somatic_mutations_GTEx). • Raw RNA-sequencing data is available through the GTEx project at dbGaP under the project id phs000424.v7.p2 (https://www.ncbi.nlm.nih.gov/projects/gap/cgi-bin/study.cgi?study_id=phs000424.v7.p2). • Chromatin data analyzed in this study is available at the Roadmap Epigenomics project (https://www.ncbi.nlm.nih.gov/projects/gap/cgi-bin/study.cgi?study_id=phs000424.v7.p2). • Mutations previously observed in cancer are available through the COSMIC v86 database (https://cosmic-blog.sanger.ac.uk/cosmic-release-v86/). • The list of cancer driver genes is available as supplementary information from a TCGA publication [2] (Additional file 13: Table S10). • All processed datasets related to somatic mutations obtained from our mutation calling method are included in this published article.
