# Supplementary material for: The somatic mutation landscape of the human body
Source: Genome Biol. 2019 Dec 24;20:298. doi: 10.1186/s13059-019-1919-5 (PMC6930685; doi:10.1186/s13059-019-1919-5)
Supplement: Supplementary file 1 — Additional file 1: Figure S1. Statistics associated to a method for calling DNA mutations from RNA-seq data. Figure S2. Calling of somatic DNA-mutations in the GTEx cohort. Figure S3. Mutation load across different mutation types in non-disease human tissues. Figure S4. Phenotypic associations and properties of mutation load in the human body. Figure S5. Number of stem cell divisions correlates weakly with mutation load in human tissues. Figure S6. Mutation profiles cluster by tissue. Figure S7. Inter-tissue mutational strand asymmetry correlations and cell type associations. Figure S8. Gene expression associations with C>T mutation load. Figure S9. Gene expression associations with overall mutation load. Figure S10. Mutation load associations with expression of genes involved in DNA repair or DNA mutagenesis. Figure S11. Negative controls and filters for cancer mutation enrichment in non-disease human tissues. Figure S12. Comparison of mutation calls to those from Yizhak et al. [file 13059_2019_1919_MOESM1_ESM.pdf]

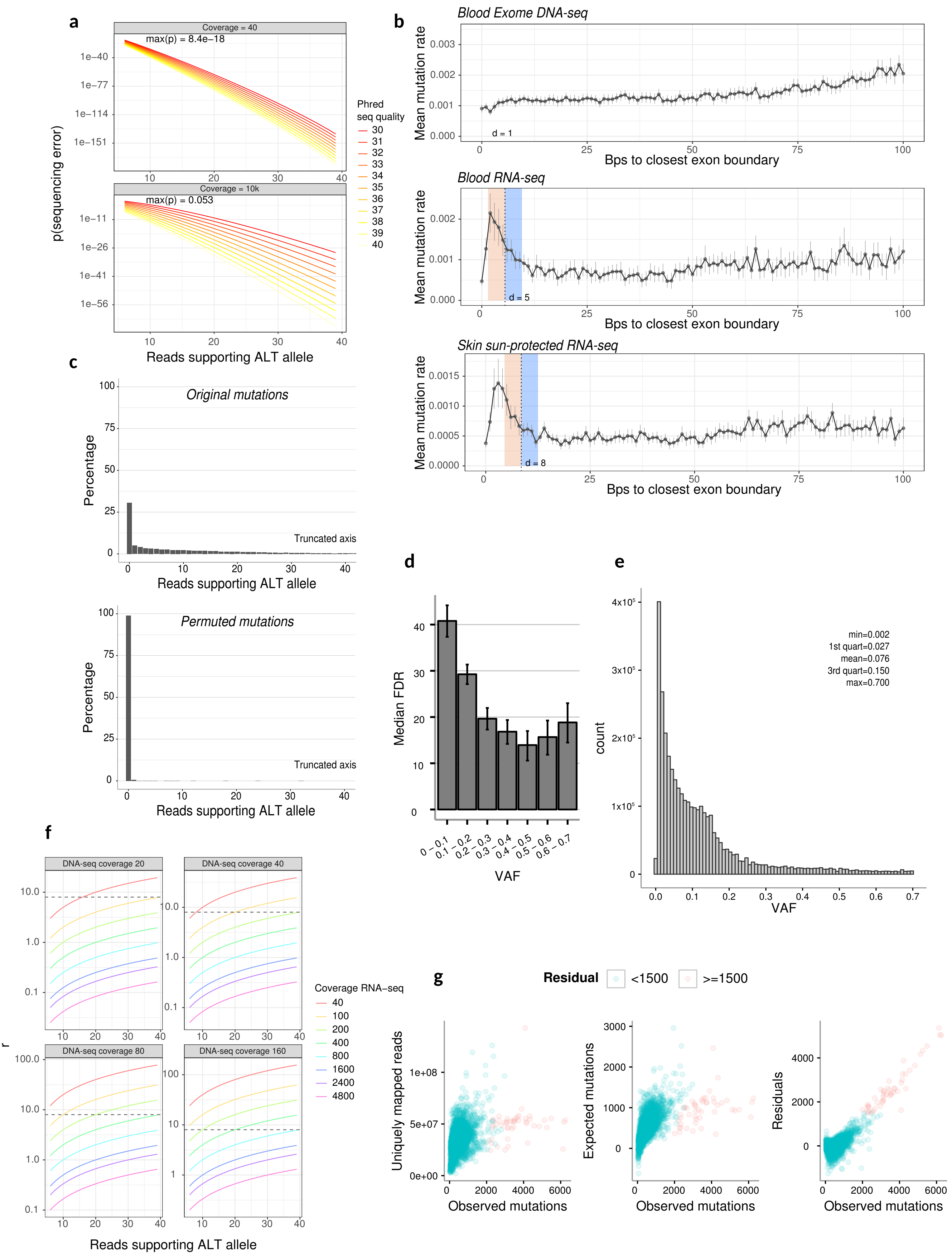
a
Coverage = 40
max(p) = 8.4e−18
1e−40
1e−77
1e−114
1e−151
Coverage = 10k
max(p) = 0.053
1e−11
1e−26
1e−41
1e−56
p(sequencing error)
Reads supporting ALT allele
Phred seq quality
30
31
32
33
34
35
36
37
38
39
40
b
Blood Exome DNA-seq
Mean mutation rate
d = 1
Bps to closest exon boundary
Blood RNA-seq
d = 5
Skin sun-protected RNA-seq
d = 8
c
Original mutations
Percentage
Truncated axis
Reads supporting ALT allele
Permuted mutations
d
Median FDR
0 − 0.1
0.1 − 0.2
0.2 − 0.3
0.3 − 0.4
0.4 − 0.5
0.5 − 0.6
0.6 − 0.7
VAF
e
count
min=0.002
1st quart=0.027
mean=0.076
3rd quart=0.150
max=0.700
VAF
f
DNA-seq coverage 20
DNA-seq coverage 40
DNA-seq coverage 80
DNA-seq coverage 160
r
Reads supporting ALT allele
Coverage RNA−seq
40
100
200
400
800
1600
2400
4800
g
Residual
<1500
>=1500
Uniquely mapped reads
Expected mutations
Residuals
Observed mutations

Fig. S1. Statistics associated to a method for calling DNA mutations from RNA-seq data. a, Probabilities of observing a sequencing error (y axis) on a position covered with the lowest coverage used in this study (top panel, 40 reads), or a highly covered base (bottom panel, 10^4 reads), with increasing number of reads supporting the alternate allele (x axis), at different phred sequencing scores (colored lines). Probability is calculated using the right tail of a binomial distribution on the number of observed reads supporting a mutation [X ~ binom(n = coverage, p=$10^{-phred/10}$x1/3); Phred-based sequencing error probability is multiplied by 1/3 to account for three potential different bases to mutate]. **b,** Average mutation rate is high close to splice junctions in RNA-seq (bottom 2 rows) but not in DNA-seq data (top row). Mutation rate was calculated by taking the number of mutations observed at a given distance from an exon junction and dividing it by the number of reads covering positions at that distance. Vertical dashed lines represent the point of inflection at which mutation rate stabilizes; we used a 1-bp sliding window to identify the position with maximum absolute difference between mutation rates of the four bps downstream and the four bps upstream of that position. Error bars are the 95% confidence intervals based on bootstrapping 1,000 times. **c,** DNA somatic mutations called from RNA-seq were validated by assessing the percentage of mutations for which there was at least one read supporting the alternate allele in matched exome DNA-seq data (see Methods). The top panel shows the histogram of number of reads supporting the alternate allele in DNA-seq for all mutations found in RNA-seq. The bottom panel shows the same data after randomly assigning an alternate allele to the mutations called in RNA-seq, effectively creating a distribution expected by chance (see Methods). X axes were truncated for visual purposes **d,** FDR for mutations of different VAFs in the RNA-seq calls (similar to Fig.1d). For 105 individuals we compared variant calls from exome DNA-seq data with those from RNA-seq of the same samples. Median FDR values are shown and they represent the fraction of mutations called in RNA-seq for which there are no exome reads supporting the same variant (see Methods and Supplementary Figure 1c). Error bars represent the 95% confidence interval after bootstrapping 10,000 times. **e,** Variant allele frequency distribution across all mutations. **f,** For the method validation, we compared RNA-seq-based mutation calls to DNA-seq data. To address differences in coverage between the two methods we only compared positions with $r \geq 8$ (dashed line; r effectively represents the number of expected reads that support the alternate allele in DNA-seq at a position given the alternate allele frequency observed in RNA-seq at that position and the DNA-seq coverage; see Methods), which ensures considering only positions for which reads supporting the alternate allele were expected to be found in DNA-seq given the coverage of that position in both experiments. **g,** Hyper-mutated samples were identified by applying a linear regression between the number of mutations and sequencing depth (Uniquely mapped reads), age, BMI and gender. The observed number of mutations was mostly explained by sequencing depth (left panel) and the expected number of mutations agrees for most samples with the observed number of mutations (middle panel). Hyper-mutated samples were tagged as the ones with residuals from the linear regression of ≥ 1,500, meaning they had at least 1,500 mutations more than expected (right panel; see Methods for more details).

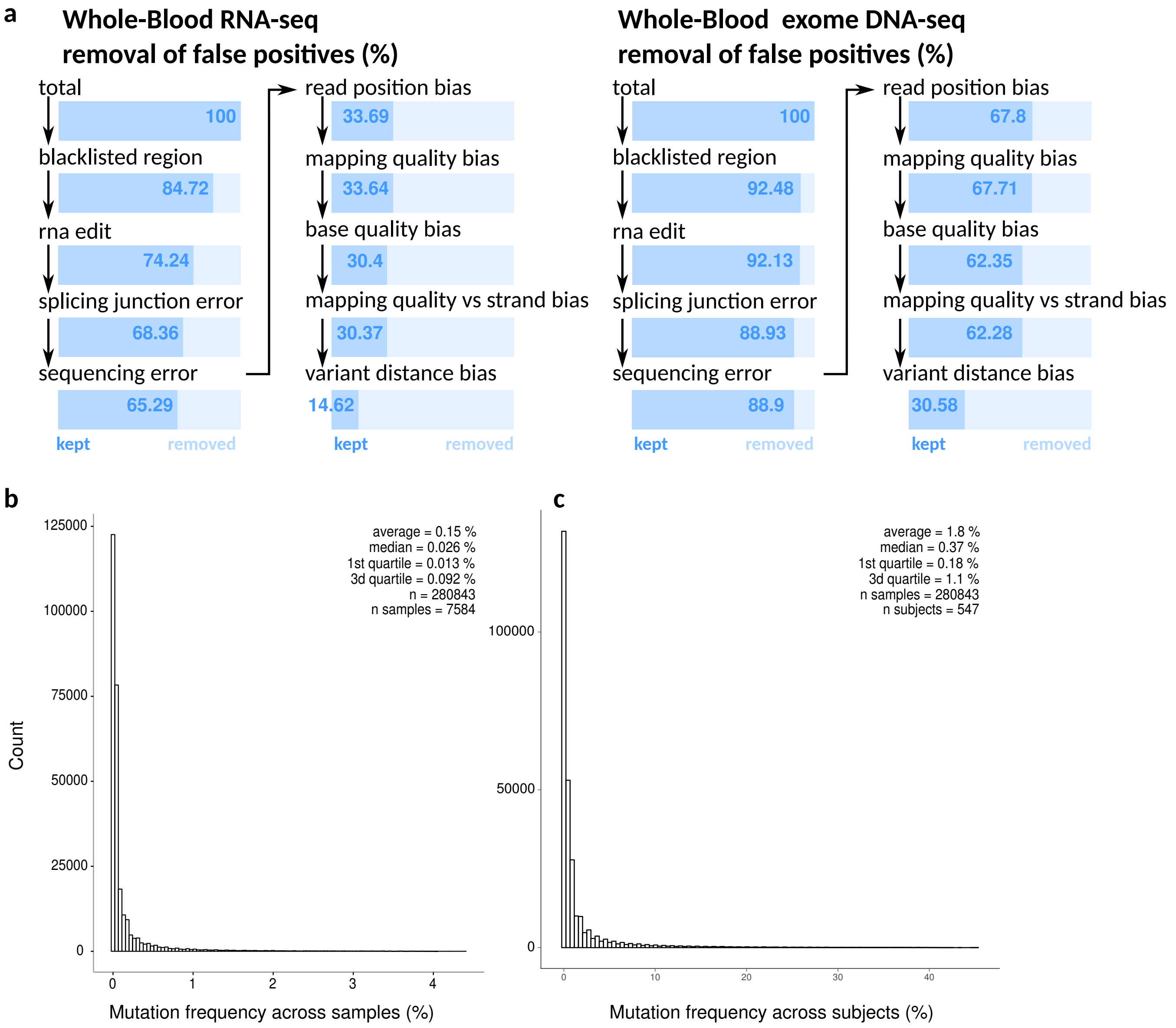

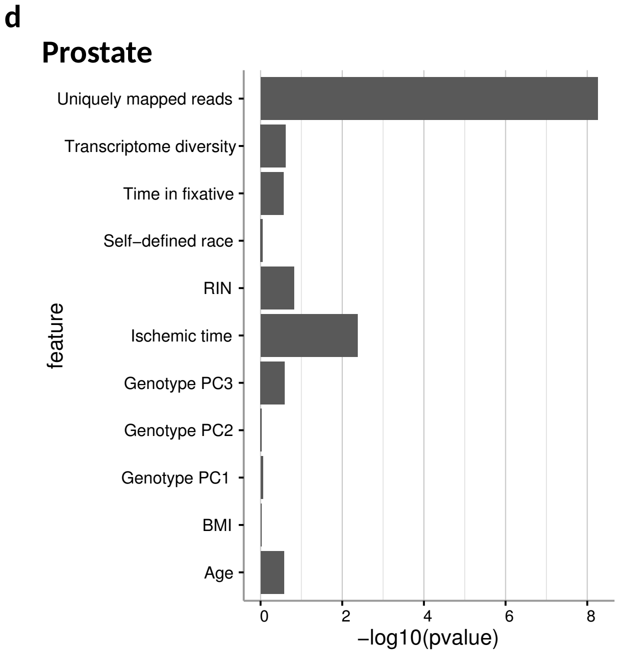

Fig. S2. Calling of somatic DNA-mutations in the GTEx cohort. a, Per-sample filters applied to mutation calls in blood RNA-seq (left) and DNA-seq (right) (For all tissue data see Additional File 3: Table S1). **b-c,** Frequency histogram in percent units of unique mutations across samples (b) and across individuals (c) **d,** Significance of associations between total number of mutations observed in prostate with biological and technical variables. P-values were obtained from a linear regression (see Methods; see Supp. Table 4 for all-tissue data).

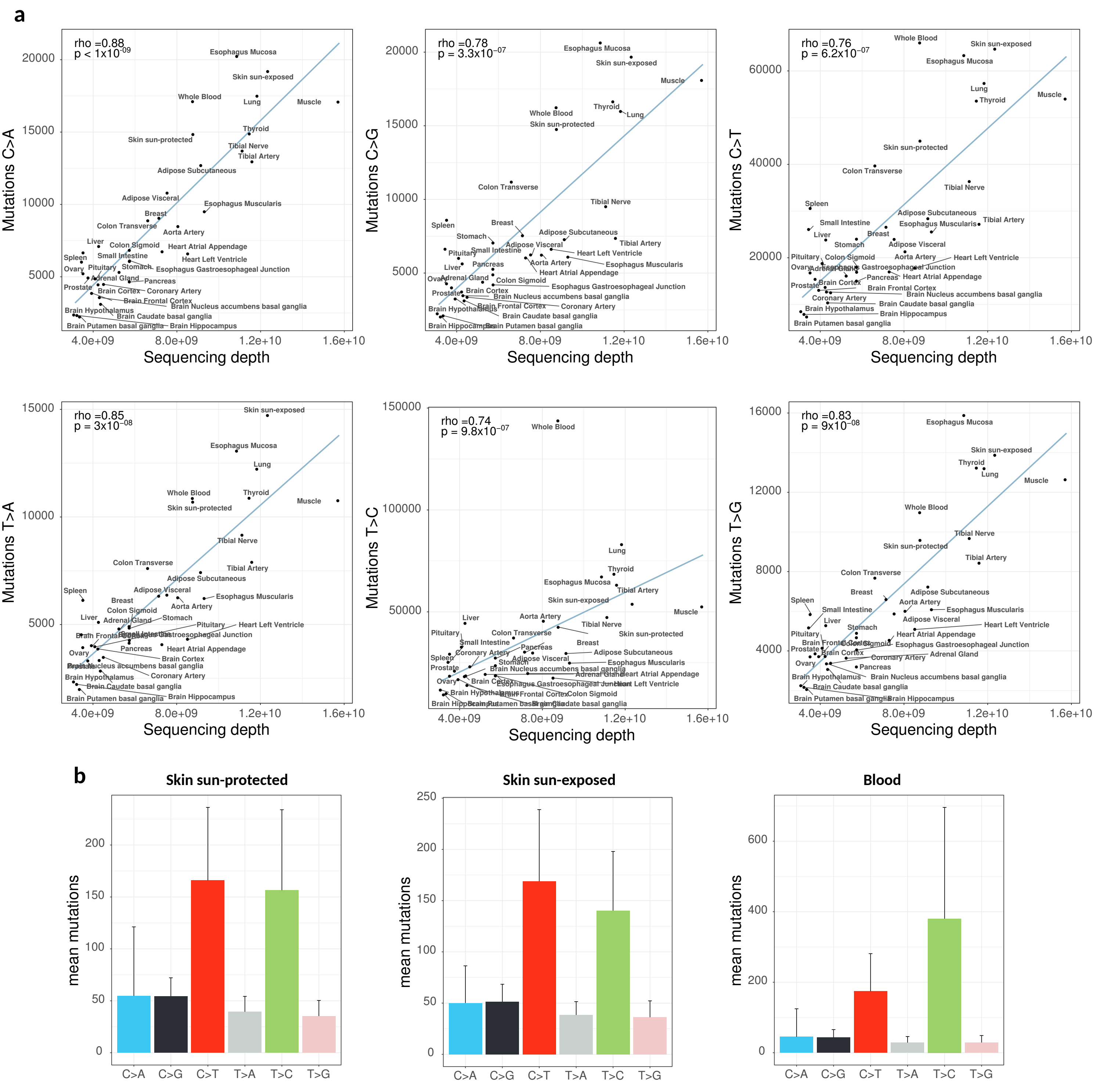

Fig. S3. Mutation load across different mutation types in non-disease human tissues. a, Across all mutation types, the total number of mutations observed in a tissue is explained by the total sequencing depth of that tissue. A linear regression line is shown in blue; tissues above it exhibit more mutations than expected by sequencing depth and tissues below it show fewer mutations than expected. *Rho* is the Spearman coefficient. **b,** Representative examples for relative contributions of different mutation types across different tissues (for all-tissue data see Additional File 7: Table S5).

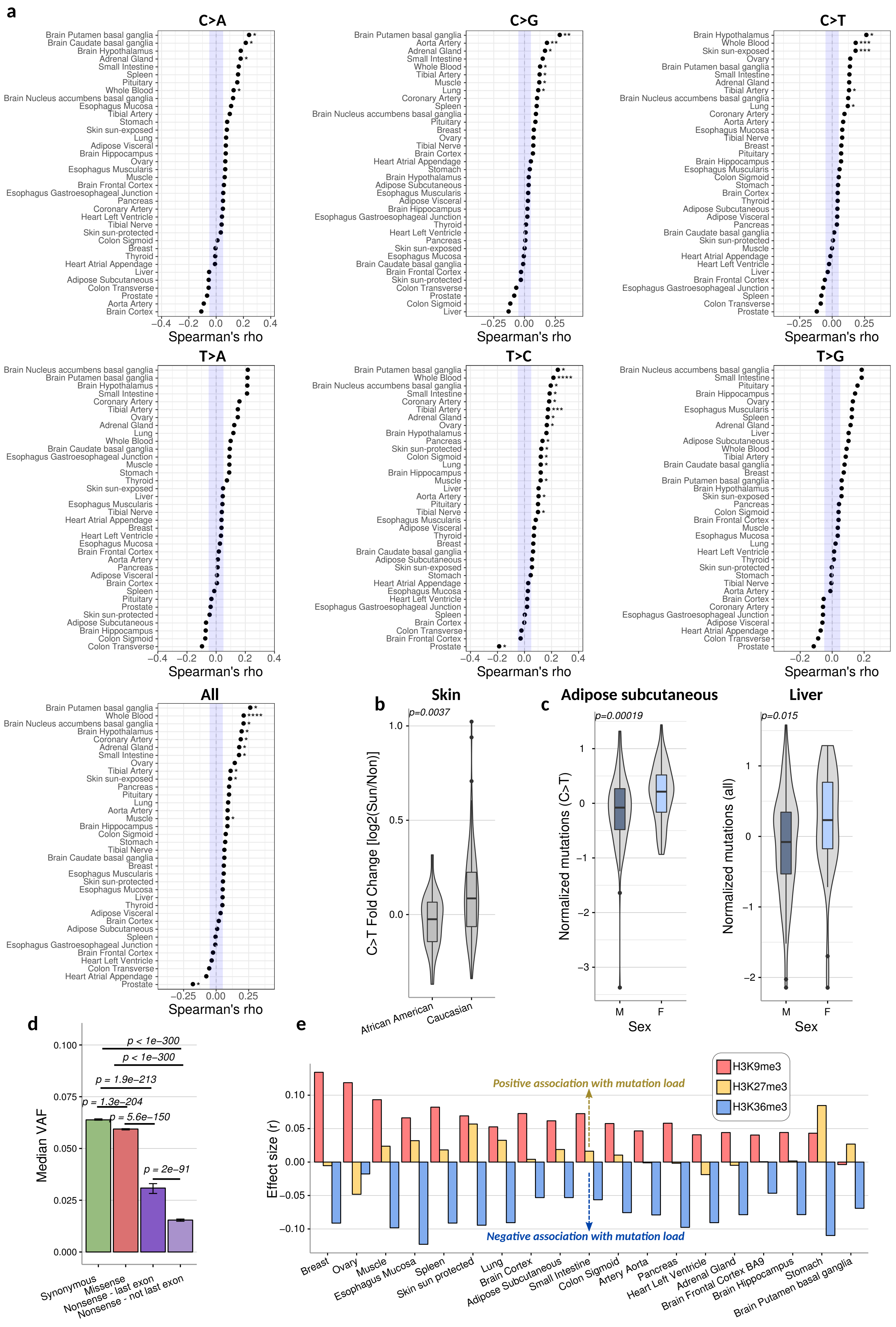

a
C>A
Brain Putamen basal ganglia
Brain Caudate basal ganglia
Brain Hypothalamus
Adrenal Gland
Small Intestine
Spleen
Pituitary
Whole Blood
Brain Nucleus accumbens basal ganglia
Esophagus Mucosa
Tibial Artery
Stomach
Skin sun-exposed
Lung
Adipose Visceral
Brain Hippocampus
Ovary
Esophagus Muscularis
Muscle
Brain Frontal Cortex
Esophagus Gastroesophageal Junction
Pancreas
Coronary Artery
Heart Left Ventricle
Tibial Nerve
Skin sun-protected
Colon Sigmoid
Breast
Thyroid
Heart Atrial Appendage
Liver
Adipose Subcutaneous
Colon Transverse
Prostate
Aorta Artery
Brain Cortex
Spearman's rho
C>G
Brain Putamen basal ganglia
Aorta Artery
Adrenal Gland
Small Intestine
Whole Blood
Tibial Artery
Muscle
Lung
Coronary Artery
Spleen
Brain Nucleus accumbens basal ganglia
Pituitary
Breast
Ovary
Tibial Nerve
Brain Cortex
Heart Atrial Appendage
Stomach
Brain Hypothalamus
Adipose Subcutaneous
Esophagus Muscularis
Adipose Visceral
Brain Hippocampus
Esophagus Gastroesophageal Junction
Thyroid
Heart Left Ventricle
Pancreas
Skin sun-exposed
Esophagus Mucosa
Brain Caudate basal ganglia
Brain Frontal Cortex
Skin sun-protected
Colon Transverse
Prostate
Colon Sigmoid
Liver
Spearman's rho
C>T
Brain Hypothalamus
Whole Blood
Skin sun-exposed
Ovary
Brain Putamen basal ganglia
Small Intestine
Adrenal Gland
Tibial Artery
Brain Nucleus accumbens basal ganglia
Lung
Coronary Artery
Aorta Artery
Esophagus Mucosa
Tibial Nerve
Breast
Pituitary
Brain Hippocampus
Esophagus Muscularis
Colon Sigmoid
Stomach
Brain Cortex
Thyroid
Adipose Subcutaneous
Adipose Visceral
Pancreas
Brain Caudate basal ganglia
Skin sun-protected
Muscle
Heart Atrial Appendage
Heart Left Ventricle
Liver
Brain Frontal Cortex
Esophagus Gastroesophageal Junction
Spleen
Colon Transverse
Prostate
Spearman's rho
T>A
Brain Nucleus accumbens basal ganglia
Brain Putamen basal ganglia
Brain Hypothalamus
Small Intestine
Coronary Artery
Tibial Artery
Ovary
Adrenal Gland
Lung
Whole Blood
Brain Caudate basal ganglia
Esophagus Gastroesophageal Junction
Muscle
Stomach
Thyroid
Skin sun-exposed
Liver
Esophagus Muscularis
Tibial Nerve
Heart Atrial Appendage
Breast
Heart Left Ventricle
Esophagus Mucosa
Brain Frontal Cortex
Aorta Artery
Pancreas
Adipose Visceral
Brain Cortex
Spleen
Pituitary
Prostate
Skin sun-protected
Adipose Subcutaneous
Brain Hippocampus
Colon Sigmoid
Colon Transverse
Spearman's rho
T>C
Brain Putamen basal ganglia
Whole Blood
Brain Nucleus accumbens basal ganglia
Small Intestine
Coronary Artery
Tibial Artery
Adrenal Gland
Ovary
Brain Hypothalamus
Pancreas
Skin sun-protected
Colon Sigmoid
Lung
Brain Hippocampus
Muscle
Liver
Aorta Artery
Pituitary
Tibial Nerve
Esophagus Muscularis
Adipose Visceral
Thyroid
Breast
Brain Caudate basal ganglia
Adipose Subcutaneous
Skin sun-exposed
Stomach
Heart Atrial Appendage
Esophagus Mucosa
Heart Left Ventricle
Esophagus Gastroesophageal Junction
Spleen
Brain Cortex
Colon Transverse
Brain Frontal Cortex
Prostate
Spearman's rho
T>G
Brain Nucleus accumbens basal ganglia
Small Intestine
Pituitary
Brain Hippocampus
Ovary
Esophagus Muscularis
Spleen
Adrenal Gland
Liver
Adipose Subcutaneous
Whole Blood
Tibial Artery
Brain Caudate basal ganglia
Breast
Brain Putamen basal ganglia
Brain Hypothalamus
Skin sun-exposed
Pancreas
Colon Sigmoid
Brain Frontal Cortex
Muscle
Esophagus Mucosa
Lung
Heart Left Ventricle
Thyroid
Skin sun-protected
Stomach
Tibial Nerve
Aorta Artery
Brain Cortex
Coronary Artery
Esophagus Gastroesophageal Junction
Adipose Visceral
Heart Atrial Appendage
Colon Transverse
Prostate
Spearman's rho
All
Brain Putamen basal ganglia
Whole Blood
Brain Nucleus accumbens basal ganglia
Brain Hypothalamus
Coronary Artery
Adrenal Gland
Small Intestine
Ovary
Tibial Artery
Skin sun-exposed
Pancreas
Pituitary
Lung
Aorta Artery
Muscle
Brain Hippocampus
Colon Sigmoid
Stomach
Tibial Nerve
Brain Caudate basal ganglia
Breast
Esophagus Muscularis
Skin sun-protected
Esophagus Mucosa
Liver
Thyroid
Adipose Visceral
Brain Cortex
Adipose Subcutaneous
Spleen
Esophagus Gastroesophageal Junction
Brain Frontal Cortex
Heart Left Ventricle
Colon Transverse
Heart Atrial Appendage
Prostate
Spearman's rho
b
Skin
p=0.0037
C>T Fold Change [log2(Sun/Non)]
African American
Caucasian
c
Adipose subcutaneous
p=0.00019
Normalized mutations (C>T)
M
F
Sex
Liver
p=0.015
Normalized mutations (all)
M
F
Sex
d
Median VAF
p < 1e–300
p < 1e–300
p = 1.9e–213
p = 1.3e–204
p = 5.6e–150
p = 2e–91
Synonymous
Missense
Nonsense - last exon
Nonsense - not last exon
e
H3K9me3
H3K27me3
H3K36me3
Positive association with mutation load
Negative association with mutation load
Effect size (r)
Breast
Ovary
Muscle
Esophagus Mucosa
Spleen
Skin sun protected
Lung
Brain Cortex
Adipose Subcutaneous
Small Intestine
Colon Sigmoid
Artery Aorta
Pancreas
Heart Left Ventricle
Adrenal Gland
Brain Frontal Cortex BA9
Brain Hippocampus
Stomach
Brain Putamen basal ganglia

Fig. S4. Phenotypic associations and properties of mutation load in the human body. a, Age associations (x axis) with mutation load per tissue (y axis) across mutation types (panels). P-values were obtained by assessing the fraction of permutation-based *rho* values greater than the original *rho* value for positive *rho* values, or the fraction of permutation-based *rho* values smaller than the original *rho* value for negative *rho* values. A total 10,000 permutations were performed. FDRs were obtained for each panel using the Benjamini-Hochberg method: **** (FDR < 0.01), *** (FDR < 0.05), ** (FDR < 0.1), * (FDR < 0.2). **b,** Fold-change of C>T mutations between matched sun-exposed and sun-protected skin samples from the same individual and stratified by self-reported race. P-value is based on a two-sided Mann-Whitney test. **c,** Two representative examples of associations between mutation load and biological sex. To control for sequencing depth and other technical artifacts, mutation values were obtained as the residuals from a linear regression using technical features as explanatory variables (see Methods, for all significant associations with sex see Additional File 8: Table S6). **d,** Related to Figure 2d, median variant allele frequency (VAF) across all mutations for each mutation type based on their impact to the amino acid sequence; nonsense mutations were divided into two different groups based on whether they are located in the last exon; error bars represent the 95% confidence interval after bootstrapping 1000 times; p-values are from two-sided Mann-Whitney tests. **e,** Related to Figure 2g, mutation load is positively associated with H3K9me3 and/or negatively associated with H3K36me3 across most tissues analyzed. Effect size (*r*) of association for individual chromatin marks was obtained from linear regressions using a given chromatin mark as explanatory variable after subtracting the effects of all other chromatin marks (semi-partial correlation, see Methods)

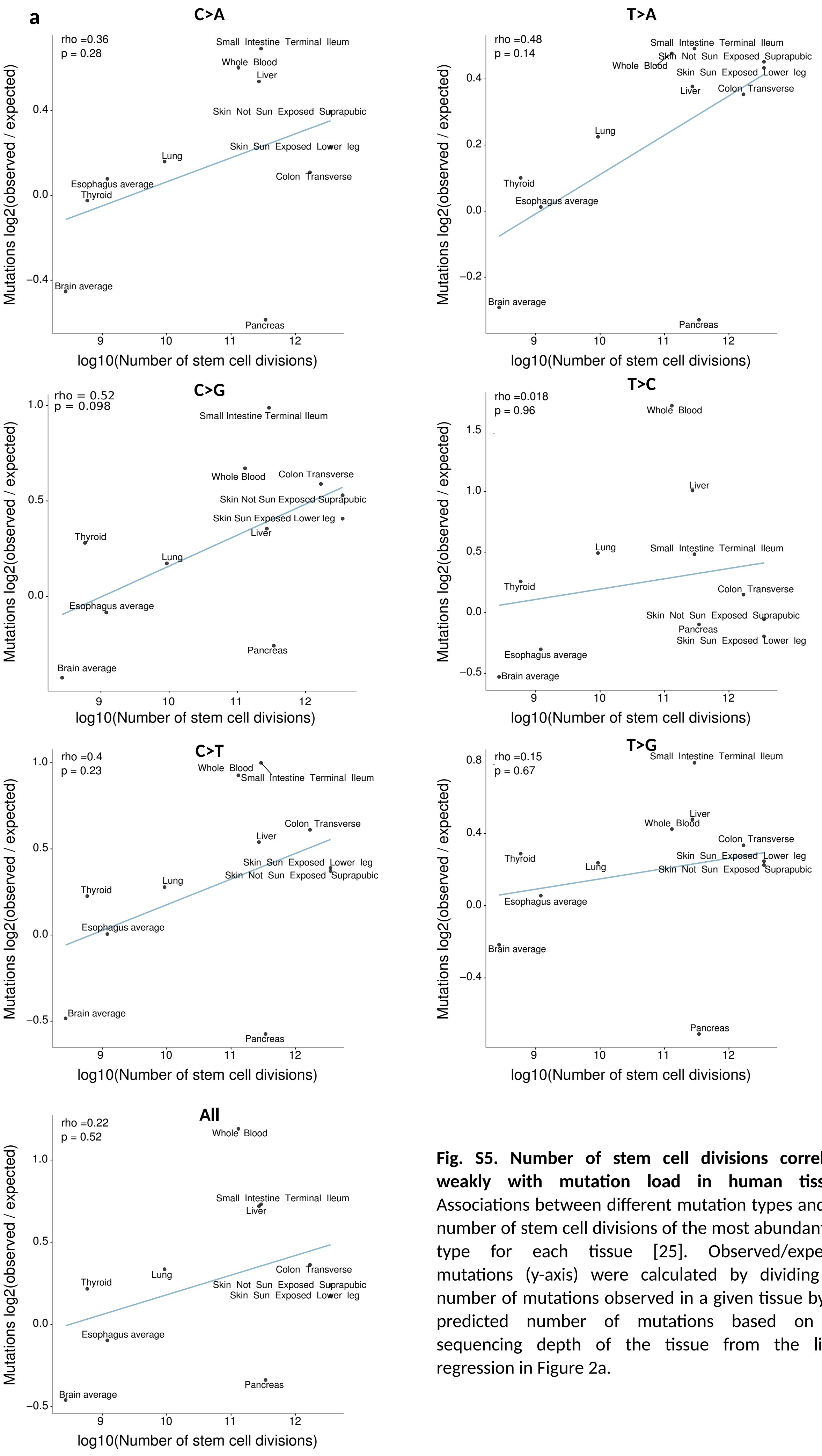

Fig. S5. Number of stem cell divisions correlates weakly with mutation load in human tissues. Associations between different mutation types and the number of stem cell divisions of the most abundant cell type for each tissue [25]. Observed/expected mutations (y-axis) were calculated by dividing the number of mutations observed in a given tissue by the predicted number of mutations based on the sequencing depth of the tissue from the linear regression in Figure 2a.

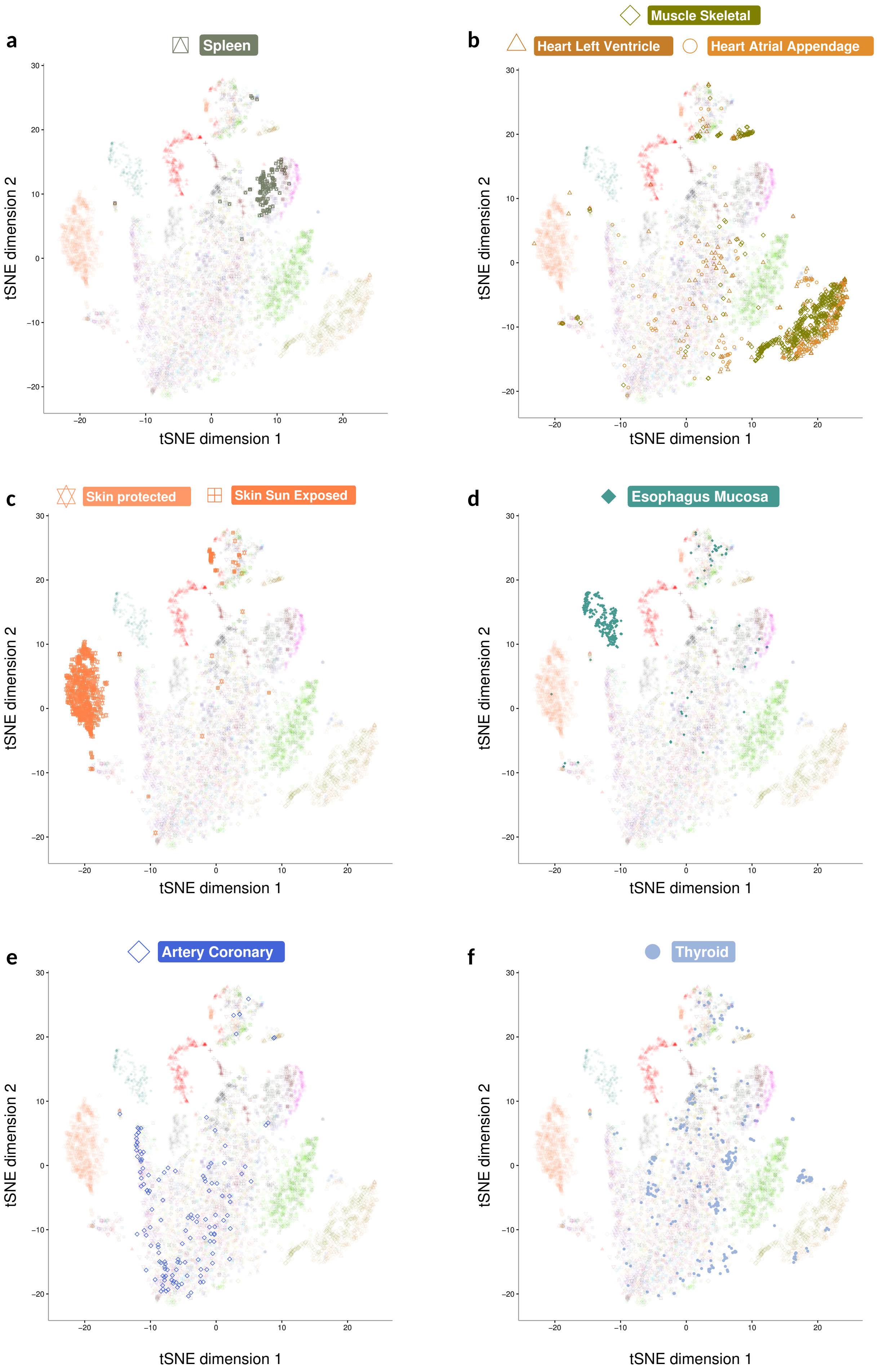

Fig. S6. Mutation profiles cluster by tissue. a-f, tSNE plots as described in Figure 2e, highlighting individual (a,d) and grouped (b,c) tissues exhibiting highly similar within-tissue mutation profiles, and two tissues showing tissue profiles with weak clustering (e,f).

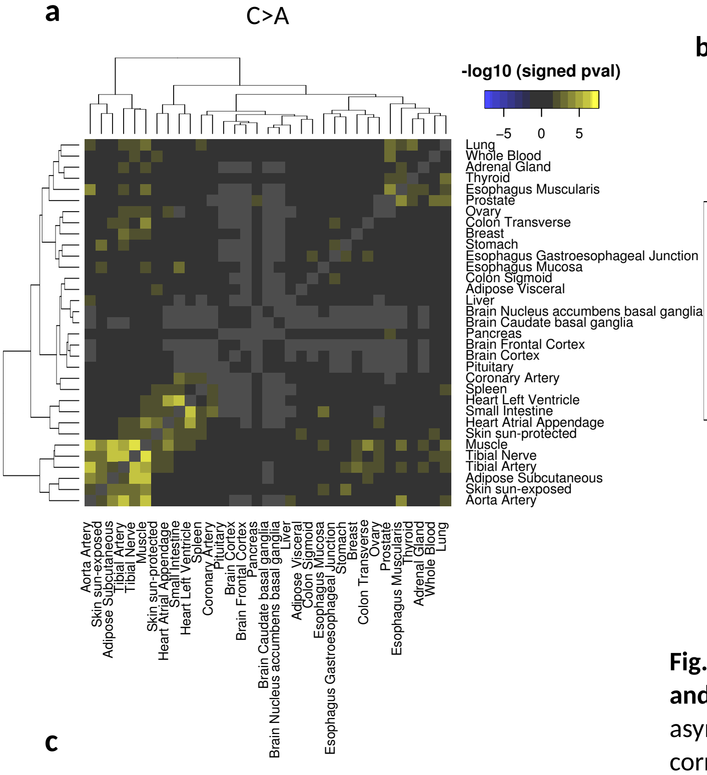

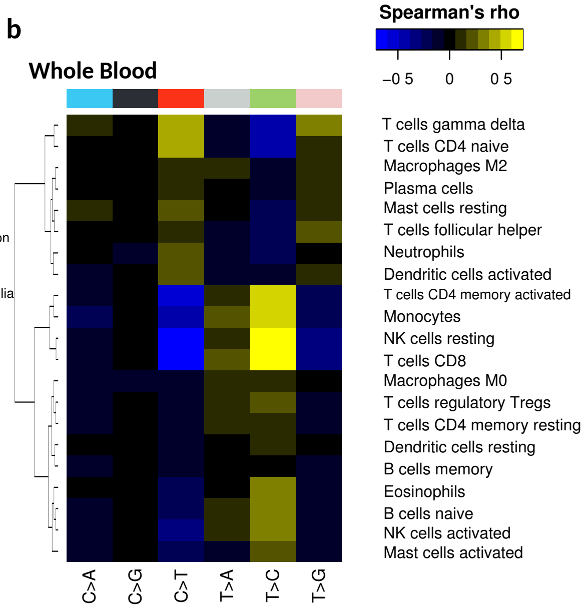

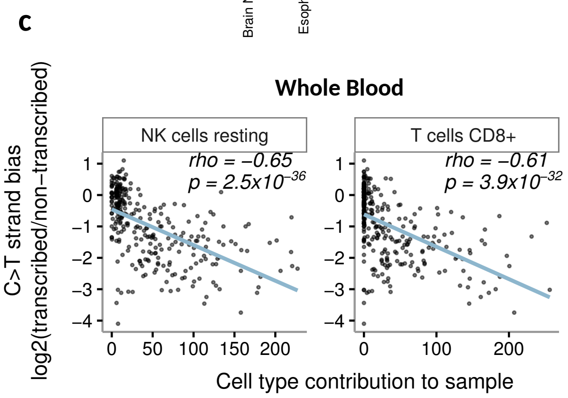

Fig. S7. Inter-tissue mutational strand asymmetry correlations and cell type associations. a, C>A inter-tissue strand asymmetry correlations; p-values are from Spearman correlations between strand asymmetry (ratio of mutations on the transcribed over the non-transcribed strand) in pairs of tissues from the same individual. Yellow indicates positive correlations, whereas blue indicates negative correlations. Tissues in light gray did not have enough samples to assess associations. Columns and rows were ordered using hierarchical clustering. **b,** Associations between C>T strand asymmetry (transcribed/non-transcribed strand) and blood cell type composition. Cell type composition was obtained using CIBERSORT [53] (see Methods). **c,** Top positive and negative correlations in blood (from panel b) between C>T strand asymmetry and cell type content.

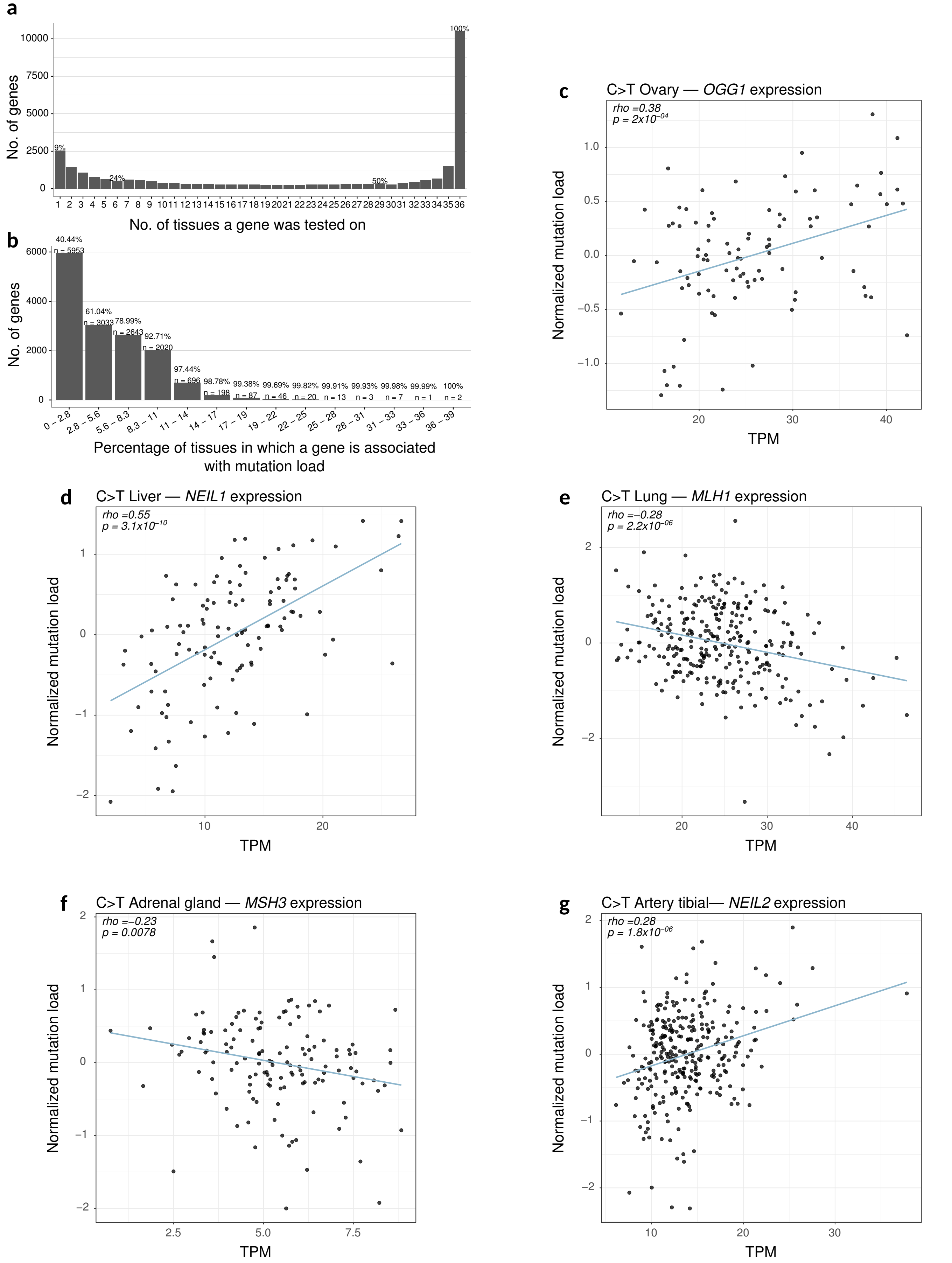

Fig. S8. Gene expression associations with C>T mutation load. a, Histogram of the number of tissues in which a gene was tested for association between its expression and C>T mutation load. A gene was selected to be tested in a tissue based on having detectable expression in that tissue (see Methods). **b,** Histogram of the percentage of tissues exhibiting significant associations ($p < 0.05$ after Bonferroni correction) between expression of a gene and C>T mutation load. **c-g,** Examples of individual significant associations between C>T mutation load and expression of DNA repair genes in different tissues ($0.001 <$ FDR < 0.2, see Fig. 4c). Mutation load is normalized by controlling for biological and technical factors (see Methods). *Rho* is the Spearman correlation coefficient.

a

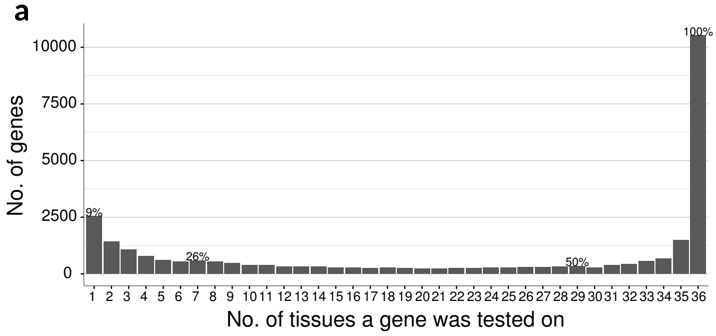

b

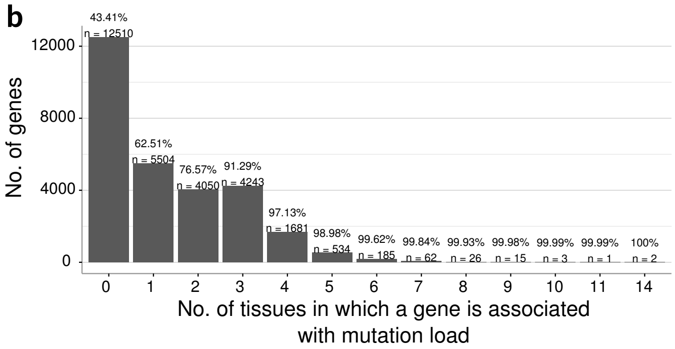

c

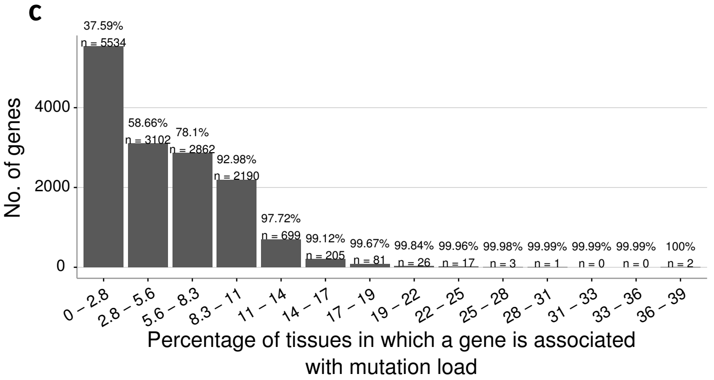

Fig. S9. Gene expression associations with overall mutation load. Similar to Figures 4a,b but for all mutation types. **a,** Histogram of the number of tissues in which each gene was detectably expressed, and thus tested for association between its expression and mutation load across all mutation types (see Methods). **b-c,** Histogram of the number (b) or percentage (c) of tissues in which a gene was significantly associated with mutation load ($p < 0.05$, Bonferroni corrected). **d,** Genes whose expression is negatively (top) or positively (bottom) associated with mutation load in multiple tissues are enriched in these representative lists of GO categories (see Methods).

d

Negative gene expression associations

Term ID	Description	FDR
GO:0048193	Golgi vesicle transport	0.00777
GO:0051641	cellular localization	0.0125
GO:0046907	intracellular transport	0.0126
GO:0007156	homophilic cell adhesion via plasma membrane adhesion molecules	0.0128
GO:0000338	protein deneddylation	0.0186
GO:0007017	microtubule-based process	0.0463
GO:0006996	organelle organization	0.0541
GO:0006289	nucleotide-excision repair	0.0608
GO:0046294	formaldehyde catabolic process	0.071
GO:0035329	hippo signaling	0.0762
GO:0016032	viral process	0.0788
GO:0015031	protein transport	0.0807

Positive gene expression associations

Term ID	Description	FDR
GO:0006629	lipid metabolic process	0.00366
GO:1901615	organic hydroxy compound metabolic process	0.00426
GO:0044281	small molecule metabolic process	0.00449
GO:0006811	ion transport	0.00565
GO:0002250	adaptive immune response	0.00567
GO:0044282	small molecule catabolic process	0.00631
GO:0051336	regulation of hydrolase activity	0.0104
GO:0006066	alcohol metabolic process	0.014
GO:0051056	regulation of small GTPase mediated signal transduction	0.0148
GO:0031424	keratinization	0.0155
GO:0046777	protein autophosphorylation	0.016
GO:1901407	regulation of phosphorylation of RNA polymerase II C-terminal domain	0.0167
GO:0008202	steroid metabolic process	0.0199
GO:0006793	phosphorus metabolic process	0.0234
GO:0007163	establishment or maintenance of cell polarity	0.0234
GO:0005975	carbohydrate metabolic process	0.0242
GO:0002376	immune system process	0.0265
GO:0046903	secretion	0.0273
GO:0019318	hexose metabolic process	0.0311
GO:0001775	cell activation	0.0463
GO:0072734	cellular response to staurosporine	0.0583
GO:0032787	monocarboxylic acid metabolic process	0.067
GO:0019216	regulation of lipid metabolic process	0.0736
GO:0090162	establishment of epithelial cell polarity	0.0786
GO:0051179	localization	0.0829
GO:0001910	regulation of leukocyte mediated cytotoxicity	0.0905
GO:0030004	cellular monovalent inorganic cation homeostasis	0.0928

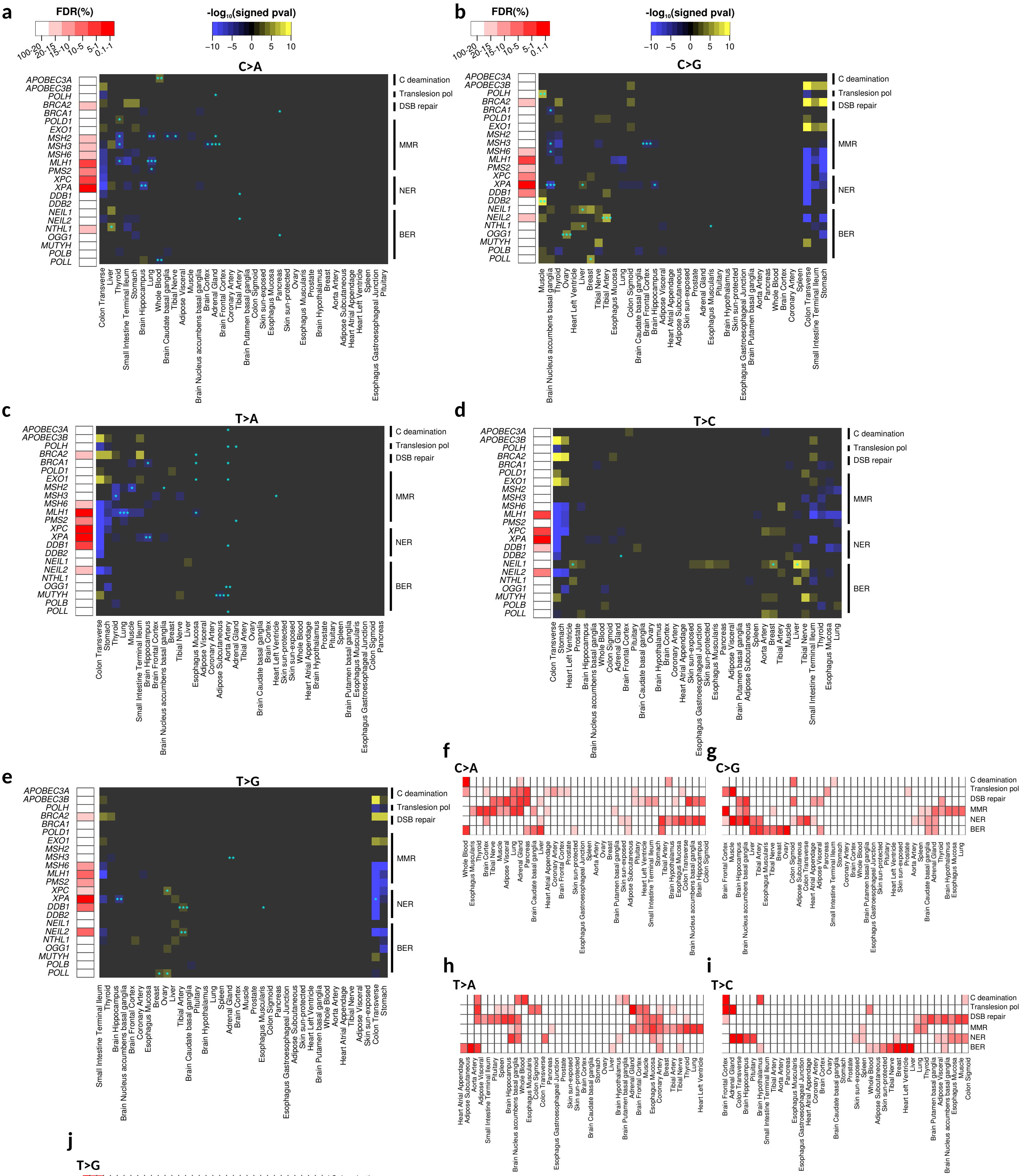

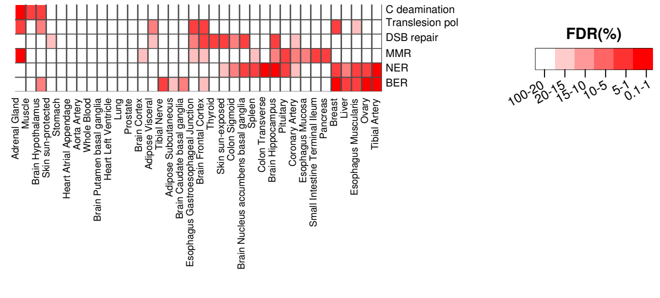

Fig. S10. Mutation load associations with expression of genes involved in DNA repair or DNA mutagenesis. a-d, Individual gene-tissue associations between different mutation types and expression of genes involved in DNA repair or DNA mutagenesis (right panels); blue stars denote significant associations using a permutation-based FDR strategy (see Methods; * FDR < 0.2, ** FDR < 0.1, *** FDR < 0.05). Shown in the left panels are genes whose expression was associated across all tissues more than expected by chance at the indicated FDR (see Methods). **e-i,** Group-level gene expression associations of the shown pathways and different mutation types across tissues (see Methods).

Supplementary Figure 11

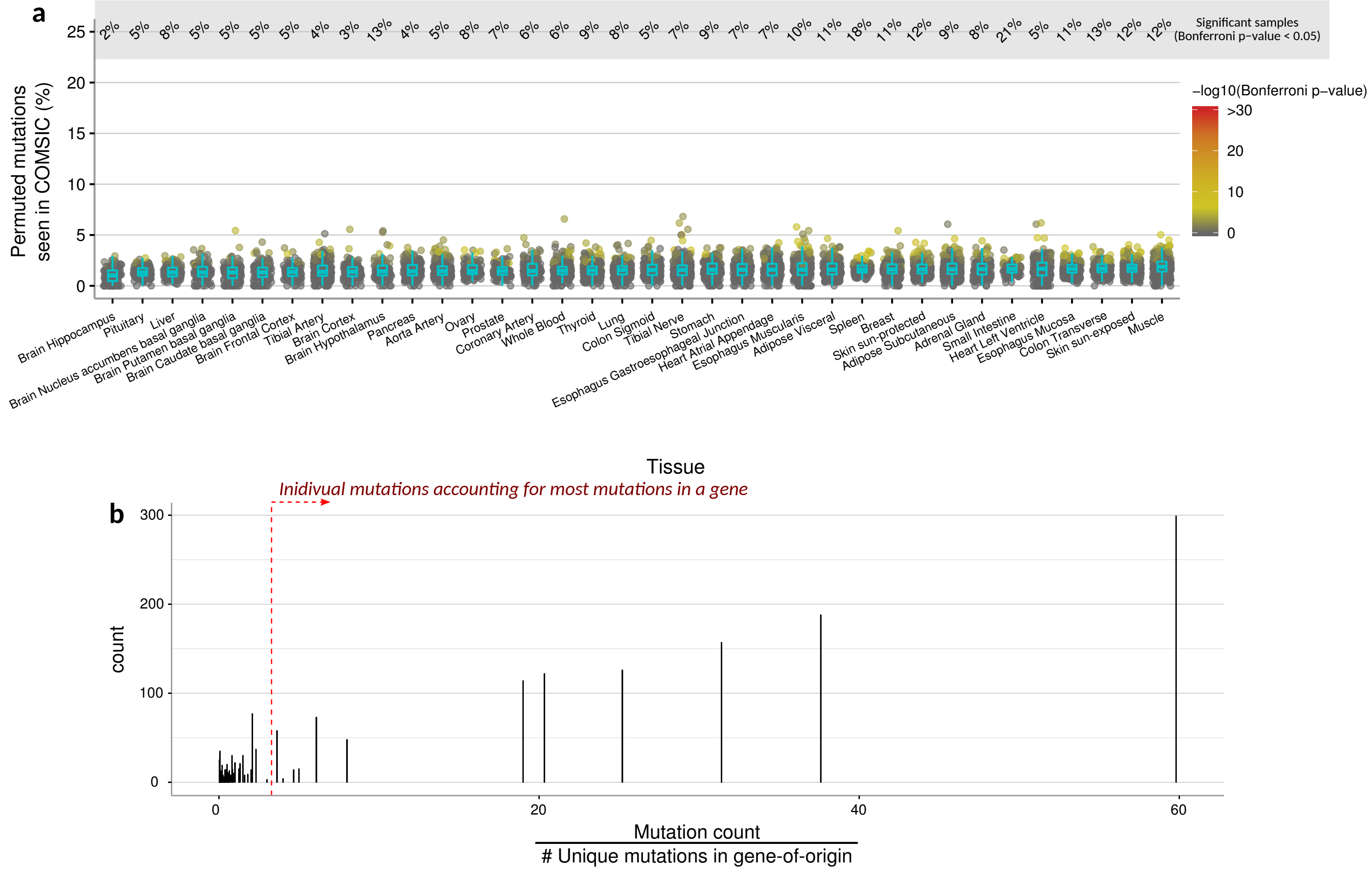

Fig. S11. Negative controls and filters for cancer mutation enrichment in non-disease human tissues. a, Percentage of randomly permuted mutations (see Methods) in non-disease tissues that overlap with cancer mutation sites (COSMIC); p-values for enrichment were calculated using a hypergeometric test accounting for sequencing coverage, total number of mutations per sample, total number of COSMIC mutations, and the three possible alternate alleles that any given reference allele can have (see Methods). P-values are Bonferroni-corrected across all samples. FDR is based on the Benjamini-Hochberg method across all samples. **b,** Histogram of t values (x axis, described in methods) for each mutation in a panel of 31 cancer driver genes. Mutations with high t values account for most of the unique mutated sites in their gene-of-origin, leading to a low diversity in mutations of a given gene. These mutations are likely systematic artifacts and can bias dN/dS ratios [9].

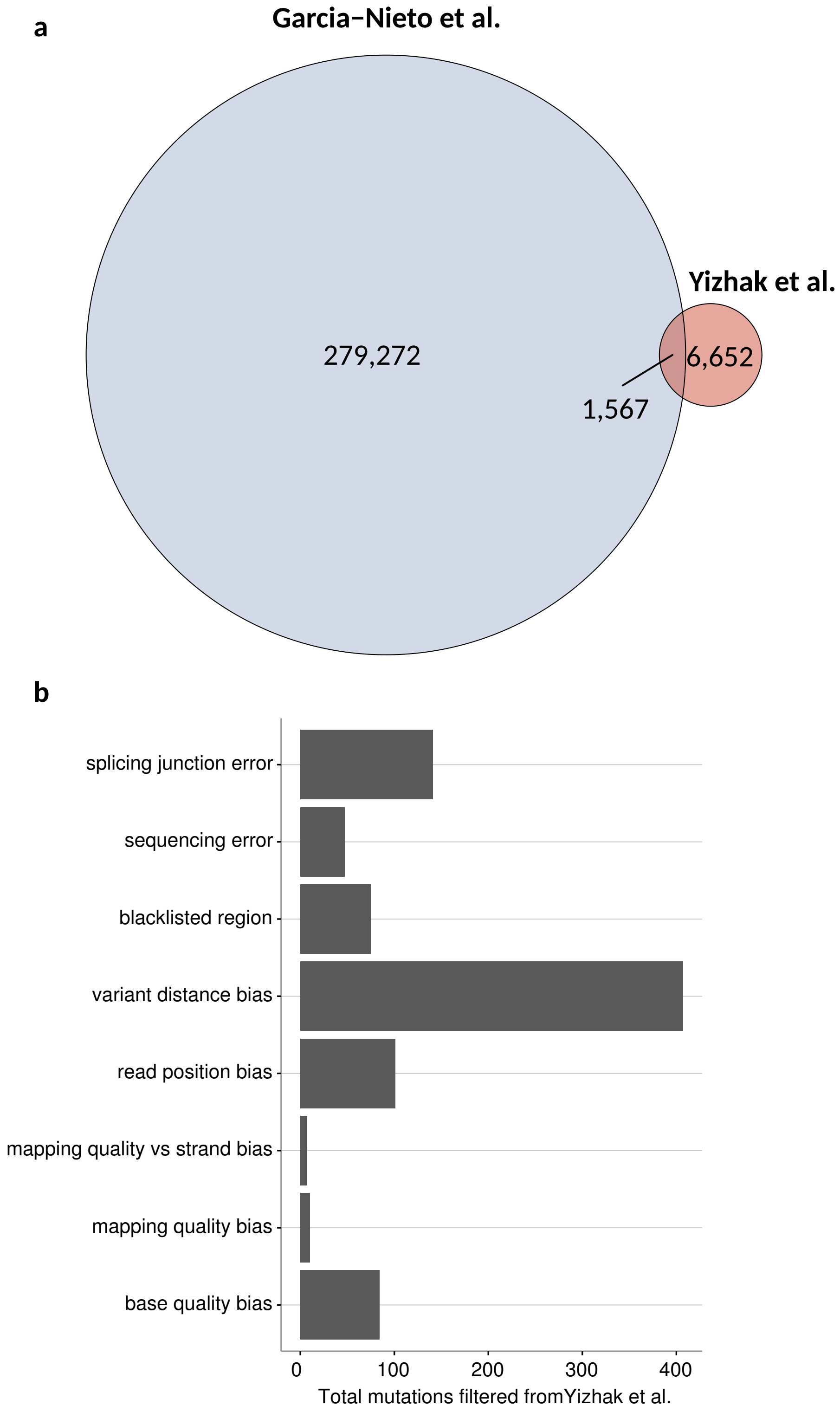

Fig. S12. Comparison of mutation calls to those from Yizhak et al. a, Venn diagram showing the total number of unique mutations found in this study and their overlap with those from a recently published study [45]. The overlap is small, as expected given the ~82% FDR of mutations called in [45], which means that we would expect no more than ~18% of this catalog to overlap with another data set (assuming false positive calls are unlikely to overlap). **b,** 632 mutations in the aforementioned study were flagged by at least one of these filters in our pipeline. The bar plot represents the number of times that a mutation was flagged by the indicated filter. We only compared mutations in the corresponding tissues between Yizhak et al. and this study.
