# Supplementary material for: The somatic mutation landscape of the human body
Source: Genome Biol. 2019 Dec 24;20:298. doi: 10.1186/s13059-019-1919-5 (PMC6930685; doi:10.1186/s13059-019-1919-5)
Supplement: Supplementary file 2 — Additional file 2: Legends for Tables S1–S13. (PDF 40 kb) [file 13059_2019_1919_MOESM2_ESM.pdf]

SUPPLEMENTARY TABLES

Table S1. Average percentage elimination of putative mutation calls by per-sample false-positive filters across all tissues.

Table S2. Total number of samples per tissue included for the final set of mutation calls.

Table S3. List of all somatic mutations identified in this study.

Table S4. P-values ($-\log_{10}$[p-value]) for the coefficients of each feature used in a linear regression on the total number of mutations per tissue.

Table S5. Average percentage of each mutation type across samples of the given tissue.

Table S6. Significant associations between biological sex and mutation load across tissues and mutation types.

Table S7. Tissue correspondence between the GTEx [1] and Roadmap Epigenomics projects [2].

Table S8. Significant GO enrichments for genes whose expression is significantly and negatively associated with C>T mutation load across several tissues.

Table S9. Significant GO enrichments for genes whose expression is significantly and positively associated with C>T mutation load across several tissues.

Table S10. List of cancer driver genes used in this study [3].

Table S11. List of mutations in cancer driver genes after further elimination of potential false positives.

Table S12. List of mutations in cancer driver genes annotated in Oncokb [4].

Table S13. Correspondence between GTEx [1] tissues and cancer types from Tomasetti and Vogelstein [5].

References

1. Aguet F, Brown AA, Castel SE, Davis JR, He Y, Jo B, et al. Genetic effects on gene expression across human tissues. Nature. 2017;550:204–13.

2. Kundaje A, Meuleman W, Ernst J, Bilenky M, Yen A, Heravi-Moussavi A, et al. Integrative analysis of 111 reference human epigenomes. Nature. 2015;518:317–30.

3. Bailey MH, Tokheim C, Porta-Pardo E, Sengupta S, Bertrand D, Weerasinghe A, et al. Comprehensive Characterization of Cancer Driver Genes and Mutations. Cell. 2018;173:371-385.e18.

4. Chakravarty D, Gao J, Phillips S, Kundra R, Zhang H, Wang J, et al. OncoKB: A Precision Oncology Knowledge Base. JCO Precis Oncol. 2017;1:1–16.

5. Tomasetti C, Vogelstein B. Variation in cancer risk among tissues can be explained by the

number of stem cell divisions. Science (80-). 2015;347:78–81.
