# Supplementary material for: The somatic mutation landscape of the human body
Source: Genome Biol. 2019 Dec 24;20:298. doi: 10.1186/s13059-019-1919-5 (PMC6930685; doi:10.1186/s13059-019-1919-5)
Supplement: Supplementary file 16 — Additional file 16. Review history [file 13059_2019_1919_MOESM16_ESM.docx]

Review History

**First round of review**

**Reviewer 1**

**Comments to author:**

This manuscript describes an approach to quantitatively detect somatic mutations in the expressed exome of multiple human tissues at various age levels as small clones, from the GTEx database. Mutations detected were those that had given rise to clones because the limits of detection would not allow to detect mutations of very low abundance, such as those unique to a cell. The results indicate that mutation loads increase with age and environmental exposure, and to some extent with sex. They find selection against missense and nonsense variants, as well as tissue specificity, associations with chromatin state and mutational strand asymmetries, and associations of expression levels of DNA repair pathways with mutation loads. Finally, they see enrichment of mutation in cancer driver genes as a function of exposure and tissue type. Overall this is a very well-written manuscript and I cannot find flaw in it. It is unfortunate that the authors apparently were scooped by the Yizhak et al paper in Science, in which very similar findings were presented. I do not consider this a weakness, of course, but it takes away the novelty of the work, which I consider very innovative. The authors make a good point regarding their vastly higher true positive mutation calls. I have only two comments:

1. Somatic mutations called from the transcriptome will distribute in different genes when analyzing different cell types. Therefore, such mutations are likely to be affected by potential cell-type differences in (1) transcribed gene length (which affect mutation load), (2) gene transcription level (which affect calling sensitivity), and (3) base pair composition of transcribed genes (which affect spectrum analysis). Did the authors consider to adjust for these factors throughout the entire analysis?

2. The authors write as if this is all very new, but there is in fact an entire literature on somatic mutations, originally from reporter genes, such as HPRT in humans and the transgenic reporter mouse models, for example, made by my own lab in the 1990s. Tissue specificities have been reported using these mouse models. Also the selection observed in this present paper were recently reported in our B lymphocyte paper in PNAS.

Jan Vijg, New York

**Reviewer 2**

**Comments to author:**

The manuscript "The somatic mutation landscape of the human body" by García-Nieto and colleagues describes analysis of somatic mutations from RNA-seq data generated by the GTEx project. This is a fundamental question to in biology, genomics, and human health. I applaud the authors to re-use the large data sets to address a question that was not part of the core questions asked by the original study design. I do have significant reservation about the robustness of the analysis.

Main comments:

1. Does FDR inferred from the validation data depends on VAF? Is the VAF distribution of the validation set representative of the whole call set?

2. Mutation frequency across samples: The 1st quartile is 0.013, which means that 75% of the mutations are carried by at least 100 samples. it's much higher than I would expect. What's the frequency distribution across subjects? In other words, are most of the recurrent mutations only occur within a single subject? What's the distribution after excluding known cancer driver genes? Without direct contribution to cancer-like clonal expansion, the chance of observing the same somatic mutation across subjects is very low.

3. tissue specific pattern: gene expression level determines the depth of coverage which in turn determines the power of detecting mutations. Is this issue accounted for in the tissue specific mutation pattern analysis? In other words, how much of the pattern shown in Figure 2e is the consequence of expression difference rather than mutation difference across tissues? The association with histone marks is an encouraging sign that mutation load is negatively correlated with expression level, but it will be more informative to show the effect size of the association with histone marks - currently Figure 2g only shows p-value.

Minor comment:

I can't find figure 1e?

**Authors Response**

**Point-by-point responses to the reviewers’ comments:**

Reviewer #2: The manuscript "The somatic mutation landscape of the human body" by García- Nieto and colleagues describes analysis of somatic mutations from RNA-seq data generated by the GTEx project. This is a fundamental question to in biology, genomics, and human health. I applaud the authors to re-use the large data sets to address a question that was not part of the core questions asked by the original study design.

*We appreciate the reviewer’s note on the importance of the questions we are addressing.*

I do have significant reservation about the robustness of the analysis. Main comments:

1.	Does FDR inferred from the validation data depends on VAF?

*We have analyzed the relationship between VAF and FDR, and have summarized the results in a new figure (Supp Fig 1d). The figure shows that FDR does depend on VAF, with low-frequency variants having a higher FDR than high-frequency variants. This is to be expected, since rare variants are less likely to be observed in an independent data set (such as the exome data we used for validation) due to their rarity. We have used this result to calculate a VAF-corrected FDR (see below).*

*We have mentioned this result in the main text: “Mutations with a higher variant allele frequency (VAF) had an overall lower FDR (Supp. Fig. 1d)…”*

Is the VAF distribution of the validation set representative of the whole call set?

*We appreciate this question, which led us to estimate a new FDR for our whole call set. The answer is that the validation set is shifted towards higher VAF than the whole call set, due to the way that we filtered out mutation calls with low power for validation. More specifically, as explained in the Methods section, for a given sample we filtered out mutations with low power for validation using the following formula:*

*Where Ci and Ai are total and alternate read counts for position i; ri effectively represents the number of reads expected to support the alternate allele in DNA-seq at position i. We took a conservative approach and only included genomic positions where r ≥ 8. Without this filter, many of the mutations being tested for validation would be so rare that zero DNA-seq reads would be expected to support them, even if the mutation were real.*

*Since this method will inherently select mutations in a way that shifts the VAF distribution towards higher values, we have estimated a VAF-corrected FDR by binning all validation variants by VAF (as in Supp Fig 1d), and then calculating an average FDR weighted by the fraction of all mutation calls present in each bin. For example, if 20% of mutations had a VAF-matched FDR of 25% and 80% of mutations had a VAF-matched FDR of 30%, then the overall FDR would be 29%.*

*Applying this approach, our VAF-corrected FDR was 34%. We have noted this in the main text as well as in the Methods.*

2.	Mutation frequency across samples: The 1st quartile is 0.013, which means that 75% of the mutations are carried by at least 100 samples. it's much higher than I would expect.

*We agree with the reviewer that if 75% of the mutations were carried by at least 100 samples that would be much higher than expected. We would like to highlight what seems to be a point of confusion, specifically what 0.013 means (shown in Supp. Fig 2b) – this number is actually a percentage (i.e. 0.013%) which is equivalent to 0.00013 frequency. In other words, 75% of the mutations are carried by at least 1 sample and not 100 samples. Expressed in another way, most mutations are only present in 1-2 samples.*

*We see that the percentage symbol this was not clearly indicated in the original manuscript (only being present in the figure axis label), so we have added these symbols next to each of the numbers in the inset as well.*

What's the frequency distribution across subjects?

In other words, are most of the recurrent mutations only occur within a single subject?

*We thank the reviewer for this question and now include this information in the manuscript in Supp. Fig. 2c and in the main text: “This resulted in a total of 280,843 unique mutations (Supp. Table 3), most of which were rare across samples (median frequency = 0.026% of samples; Supp. Fig. 2b) and across individuals (median frequency = 0.37% of individuals; Supp. Fig. 2c).”*

*To address the reviewer’s second question, based on this frequency distribution across subjects most mutations are rare across individuals (median = 0.37% or 2 individuals) and there are few recurrent mutations across individuals (third quartile = 1.1% or 6 individuals).*

What's the distribution after excluding known cancer driver genes? Without direct contribution to cancer-like clonal expansion, the chance of observing the same somatic mutation across subjects is very low.

*We agree that cancer mutations should be more frequent across individuals as compared to non-cancer mutations. To explore this, we separated mutations into two sets: mutations present in the COSMIC database (which contains nearly all mutations previously documented to have been observed in cancer) and mutations not present in COSMIC. We then compared the median frequency across subjects of the two sets of mutations and we observed that cancer mutations across tissues are 1.06x to 2.16x more frequent that non-cancer mutations (consistent with the reviewer’s expectation). Cancer mutations are more frequent specifically in non-brain tissues:*

*We now include this results in the main text with the following sentence: “In addition, cancer- associated mutations were found in more individuals compared to other mutations, particularly in non-brain tissues (median of 1.7-fold more individuals for non-brain mutations, and 1.2-fold for brain).”*

3.	tissue specific pattern: gene expression level determines the depth of coverage which in turn determines the power of detecting mutations. Is this issue accounted for in the tissue specific mutation pattern analysis? In other words, how much of the pattern shown in Figure 2e is the consequence of expression difference rather than mutation difference across tissues?

*We agree with the reviewer that gene expression level determines the depth of coverage which in turn influences the power of detecting mutations. Specifically, in Figure 2e (which was constructed using mutation profiles based on a pentanucleotide model, i.e. the mutation plus 2 upstream and downstream nucleotides), expression levels influence the representation of each pentanucleotide sequence and therefore affect our power to call mutations. Therefore it is critical to correct for the expression level of every possible pentanucleotide sequence when comparing mutation rates across tissues.*

*In the original manuscript, our description of this normalization process was not as clear as it could be. Therefore we have attempted to clarify this, both in the manuscript and below for the reviewer.*

*In the following hypothetical example, two mutations are shown for two different samples. The raw counts for the first mutation are different (AT[C>T]GG), however the content of the background sequence (ATCGG) is also different across samples and therefore after normalization the mutation rate is not different across samples. Conversely, using this method we can identify real mutation differences as in the case of the second mutation (TT[G>C]AC).*

*We have further explained this normalization in the corresponding Methods section to make it clear to the reader how we are accounting for the influence of expression differences across samples and tissues.*

*The association with histone marks is an encouraging sign that mutation load is negatively correlated with expression level, but it will be more informative to show the effect size of the association with histone marks - currently Figure 2g only shows p-value.*

*For our chromatin analysis we understand that showing effect sizes together with their p-values is valuable and we now include the effect sizes in Supp. Fig. 4e. We also include a reference to the figure in the main text.*

Minor comment:

I can't find figure 1e?

*We apologize for the typo; this should have been Figure 1d.*

Reviewer #1: This manuscript describes an approach to quantitatively detect somatic mutations in the expressed exome of multiple human tissues at various age levels as small clones, from the GTEx database. Mutations detected were those that had given rise to clones because the limits of detection would not allow to detect mutations of very low abundance, such as those unique to a cell. The results indicate that mutation loads increase with age and environmental exposure, and to some extent with sex. They find selection against missense and nonsense variants, as well as tissue specificity, associations with chromatin state and mutational strand asymmetries, and associations of expression levels of DNA repair pathways with mutation loads. Finally, they see enrichment of mutation in cancer driver genes as a function of exposure and tissue type. Overall this is a very well-written manuscript and I cannot find flaw in it. It is unfortunate that the authors apparently were scooped by the Yizhak et al paper in Science, in which very similar findings were presented. I do not consider this a weakness, of course, but it takes away the novelty of the work, which I consider very innovative. The authors make a good point regarding their vastly higher true positive mutation calls.

*We appreciate these kind comments.*

I have only two comments:

1.	Somatic mutations called from the transcriptome will distribute in different genes when analyzing different cell types. Therefore, such mutations are likely to be affected by potential cell-type differences in (1) transcribed gene length (which affect mutation load), (2) gene transcription level (which affect calling sensitivity), and (3) base pair composition of transcribed genes (which affect spectrum analysis). Did the authors consider to adjust for these factors throughout the entire analysis?

*We understand the reviewer’s concern of how cell-type composition and other factors can influence the transcriptome-based mutations in terms of mutation load, sensitivity and mutation spectrum. This is indeed one of the most important aspects to consider throughout our study and we therefore accounted for the possibility of transcriptome-based artifacts across our study in the following ways:*

*1.	We did not perform cross-sample comparisons of mutation load at the gene level since this type of analysis is the one to be affected to the greatest extent by all of the*

*reviewer’s concerns.*

*2.	Points 1 and 2 are important to address in sample- and tissue-level comparisons, because sample- and tissue-level coverage levels (influenced by the transcriptome) account for most of the variability in mutation load (as shown in Fig. 2a and Supp. Fig. 1f). Therefore, for all analysis that included sample- and tissue-level comparisons we accounted for coverage and transcriptome diversity by working with normalized mutation load.*

*a.	In the case of our “number of stem cell division analysis” we normalized mutations by calculating the observed/expected mutations (by dividing the number of mutations observed in a given tissue by the predicted number of mutations based on the sequencing depth of the tissue from the linear regression in Figure 2a)*

*b.	For our chromatin analysis, which is a tissue-level analysis across exons, we accounted for exon coverage differences by binning exons based on their expression level and then calculating the expected number of mutations per bin.*

*This expected number of mutations was then used to normalize the observed number of mutations of each exon.*

*c.	For sample-level analyses of association between mutation load and subject phenotypes, as well as between mutation load and gene expression, we included sample-level coverage and transcriptome diversity as covariates in our models. The p-values related to these analyses represent the significance of the associations controlling for these covariates, and all of our visual representations showed the residuals of the linear regression after accounting for both coverage and transcriptome diversity (as well as other potential artifacts shown in Supp. Fig. 2a)*

*d.	In our cancer section, we calculated enrichment of COSMIC cancer mutations in each sample by using a hypergeometric test that accounts for the total sequencing depth in each sample.*

*e.	For our analysis of cancer driver genes across different tissues (Fig. 5b), we accounted for total coverage differences across tissues by using mutation rates instead of total mutation counts.*

*3.	Concern number 3 is relevant in our mutation profile analysis (tSNE plot in Figs. 2e-f), where it is important to account for the different abundances of every possible pentanucleotide in the RNA-seq reads of each sample. For a detailed explanation of this normalization, please see our response to question 3 of reviewer #2.*

2.	The authors write as if this is all very new, but there is in fact an entire literature on somatic mutations, originally from reporter genes, such as HPRT in humans and the transgenic reporter mouse models, for example, made by my own lab in the 1990s. Tissue specificities have been reported using these mouse models. Also the selection observed in this present paper were recently reported in our B lymphocyte paper in PNAS.

*We thank the reviewer for pointing out these very relevant references. We have expanded our discussion to highlight some previous research that includes the use of gene reporters in animal models (Martus, Dolle, Gossen, Boerrigter, & Vijg, 1995), the early characterization of mutagenesis in aging (Vijg & Gossen, 1993), and the observation of somatic negative selection in B-lymphocytes (Zhang et al., 2019).*

**Second round of review**

**Reviewer 2**

The authors have addressed my previous concerns.
